# Anti-Bovine Programmed Death-1 Rat–Bovine Chimeric Antibody for Immunotherapy of Bovine Leukemia Virus Infection in Cattle

**DOI:** 10.3389/fimmu.2017.00650

**Published:** 2017-06-07

**Authors:** Tomohiro Okagawa, Satoru Konnai, Asami Nishimori, Naoya Maekawa, Ryoyo Ikebuchi, Shinya Goto, Chie Nakajima, Junko Kohara, Satoshi Ogasawara, Yukinari Kato, Yasuhiko Suzuki, Shiro Murata, Kazuhiko Ohashi

**Affiliations:** ^1^Department of Disease Control, Graduate School of Veterinary Medicine, Hokkaido University, Sapporo, Japan; ^2^Division of Bioresources, Research Center for Zoonosis Control, Hokkaido University, Sapporo, Japan; ^3^Global Station for Zoonosis Control, Global Institution for Collaborative Research and Education (GI-CoRE), Hokkaido University, Sapporo, Japan; ^4^Animal Research Center, Agriculture Research Department, Hokkaido Research Organization, Shintoku, Japan; ^5^Department of Regional Innovation, Tohoku University Graduate School of Medicine, Sendai, Japan; ^6^Department of Antibody Drug Development, Tohoku University Graduate School of Medicine, Sendai, Japan; ^7^New Industry Creation Hatchery Center, Tohoku University, Sendai, Japan

**Keywords:** immunoinhibitory molecules, programmed death-1, PD-ligand 1, T-cell exhaustion, immunotherapy, chimeric antibody, bovine leukemia virus, cattle

## Abstract

Blockade of immunoinhibitory molecules, such as programmed death-1 (PD-1)/PD-ligand 1 (PD-L1), is a promising strategy for reinvigorating exhausted T cells and preventing disease progression in a variety of chronic infections. Application of this therapeutic strategy to cattle requires bovinized chimeric antibody targeting immunoinhibitory molecules. In this study, anti-bovine PD-1 rat–bovine chimeric monoclonal antibody 5D2 (Boch5D2) was constructed with mammalian expression systems, and its biochemical function and antiviral effect were characterized *in vitro* and *in vivo* using cattle infected with bovine leukemia virus (BLV). Purified Boch5D2 was capable of detecting bovine PD-1 molecules expressed on cell membranes in flow cytometric analysis. In particular, Biacore analysis determined that the binding affinity of Boch5D2 to bovine PD-1 protein was similar to that of the original anti-bovine PD-1 rat monoclonal antibody 5D2. Boch5D2 was also capable of blocking PD-1/PD-L1 binding at the same level as 5D2. The immunomodulatory and therapeutic effects of Boch5D2 were evaluated by *in vivo* administration of the antibody to a BLV-infected calf. Inoculated Boch5D2 was sustained in the serum for a longer period. Boch5D2 inoculation resulted in activation of the proliferation of BLV-specific CD4^+^ T cells and decrease in the proviral load of BLV in the peripheral blood. This study demonstrates that Boch5D2 retains an equivalent biochemical function to that of the original antibody 5D2 and is a candidate therapeutic agent for regulating antiviral immune response *in vivo*. Clinical efficacy of PD-1/PD-L1 blockade awaits further experimentation with a large number of animals.

## Introduction

For decades, a variety of studies have attempted to enhance the T-cell response in chronic infections. However, immunoinhibitory pathways such as programmed death-1 (PD-1)/PD-ligand 1 (PD-L1) downregulate T-cell functions, likely causing the failure of previous attempts to develop vaccines and immunotherapies (1–3). Antibodies that block PD-1/PD-L1 can restore T-cell function and reduce viral load *in vivo* in mouse and non-human primate models (4–6). These antibodies are clearly potential novel therapeutic agents for the control of chronic infections. Recently, anti-human PD-1 antibodies have been approved and launched for the treatment of melanoma, non-small cell lung cancer, renal cell carcinoma, and Hodgkin’s lymphoma in humans (7–10). In addition, PD-1/PD-L1 blockade is under consideration for immunotherapy against chronic infections with human immunodeficiency virus, Epstein–Barr virus, hepatitis B virus, hepatitis C virus, and *Mycobacterium tuberculosis* (11–15) in human medicine. To date, however, blockers of PD-1/PD-L1 have not been approved for clinical use in veterinary medicine, including cattle.

Functional exhaustion of T-cell response has also been reported in cattle infected with bovine leukemia virus (BLV) (16–20), *Mycobacterium avium* subsp. *paratuberculosis* (21, 22), *Anaplasma marginale* (23), and *Mycobacterium bovis* (24). T-cell exhaustion may play a role in the immunopathogenesis of these diseases where pathogens evade immune elimination and establish persistent infection. To reveal the mechanism responsible for T-cell exhaustion in cattle, our previous studies investigated the expression and function of the bovine PD-1/PD-L1 pathway in BLV infection (25, 26), paratuberculosis (27), bovine anaplasmosis (28), and bovine mycoplasmosis (29). PD-1 is upregulated in CD4^+^ and/or CD8^+^ T cells during B-cell lymphoma caused by BLV infection (25), subclinical stage of paratuberculosis (27), acute anaplasmosis (28), and clinical mycoplasmosis (29). In contrast, the expression level of PD-L1 increases on infected cells and antigen-presenting cells, including BLV-infected B cells (25, 26), *M. avium* subsp. *paratuberculosis*-infected macrophages (27), and peripheral monocytes in anaplasmosis (28) and mycoplasmosis (29). Thus, the PD-1/PD-L1 axis is involved in the inhibition of T-cell function during disease progression in several chronic infections in cattle. Additionally, we established several clones of anti-bovine PD-1 rat monoclonal antibodies (mAbs) that are capable of blocking the interaction of PD-1 and PD-L1 and activating the functions of bovine T cells (25). More remarkably, PD-1/PD-L1 blockade using one of these blocking mAbs, clone 5D2, inhibits the expression of BLV gp51 protein and B-cell activation *in vitro* (25). Therefore, the PD-1/PD-L1 pathway is a candidate therapeutic target for chronic infections in cattle.

However, blocking antibodies derived from rat are considered to be not suitable for administration to cattle. Previous studies have shown that the administration of mouse antibody to cattle induces the bovine anti-mouse antibody response within 10–14 days in *in vivo* depletion experiments, as the mouse antibody is recognized as a heterologous protein in cattle (30–32). The immunogenicity of heterologous antibody is known to depend mainly (90%) on the constant regions (33). For this reason, replacing constant regions of heterologous antibody with those of bovine immunoglobulins is expected to reduce the bovine anti-antibody response and remain stable and effective for a longer period *in vivo* (31, 34).

In this study, we established anti-bovine PD-1 rat–bovine chimeric antibody (chAb), named as Boch5D2. Boch5D2 consists of variable regions from anti-bovine PD-1 rat mAb and constant regions from bovine IgG_1_ and Ig lambda. Additionally, amino acid residues of the constant domain of Boch5D2 IgG_1_ were mutated to reduce effector functions mediated *via* Fcγ receptors (FcγRs). We examine mammalian expression systems for the production of Boch5D2. The stable expression system was successful in producing sufficient amounts of Boch5D2 for further experiments. The purified Boch5D2 was tested for biochemical properties compared with the original anti-bovine PD-1 rat mAb 5D2. Accordingly, *in vivo* administration of anti-PD-1 antibodies 5D2 and Boch5D2 was conducted to clarify the *in vivo* stability and antiviral effects of these blocking antibodies in BLV-infected cattle.

## Materials and Methods

### Cloning of cDNA Encoding the Variable Regions of Anti-Bovine PD-1 Rat mAb

Total RNA was isolated from cultivated clones of hybridomas producing anti-bovine PD-1 rat mAb (5D2) (25) with the use of the TRIzol reagent (Thermo Fisher Scientific, Waltham, MA, USA) according to the manufacturer’s instructions. cDNAs encoding the variable regions of rat immunoglobulin, IgG_2a_, and Igk were amplified with a 5′-Rapid Amplification of cDNA Ends (5′-RACE) System (Thermo Fisher Scientific). Briefly, first strand cDNAs were synthesized from the obtained total RNA with a rat IgG_2a_-specific primer (RACE RAG2a-1) and a rat Igk-specific primer (RACE RACK-1) (35). Primer sequences are presented in Table S1 in Supplementary Material. After removal of the RNA template by RNase and purification of the first strand product by S.N.A.P. column (Thermo Fisher Scientific), the obtained cDNAs were tailed with poly(C) on their 3′-ends and further amplified using poly(G) primer and the other gene-specific primers, RACE RAG2a-2 (rat IgG_2a_) and RACE RACK-2 (rat Igk) (35). The polymerase chain reaction (PCR) amplicons were purified with a FastGene Gel/PCR Extraction Kit (Nippon Genetics, Tokyo, Japan) and cloned into the TA cloning site of pGEM-T Easy Vector (Promega, Madison, WI, USA). The plasmid clones were purified with a Plasmid DNA Purification Kit (Qiagen, Hilden, Germany) and sequenced with a CEQ 2000 DNA Analysis System (Beckman Coulter, Fullerton, CA, USA).

### Expression of Boch5D2 in CHO DG44 Cells

**Figure 1:**
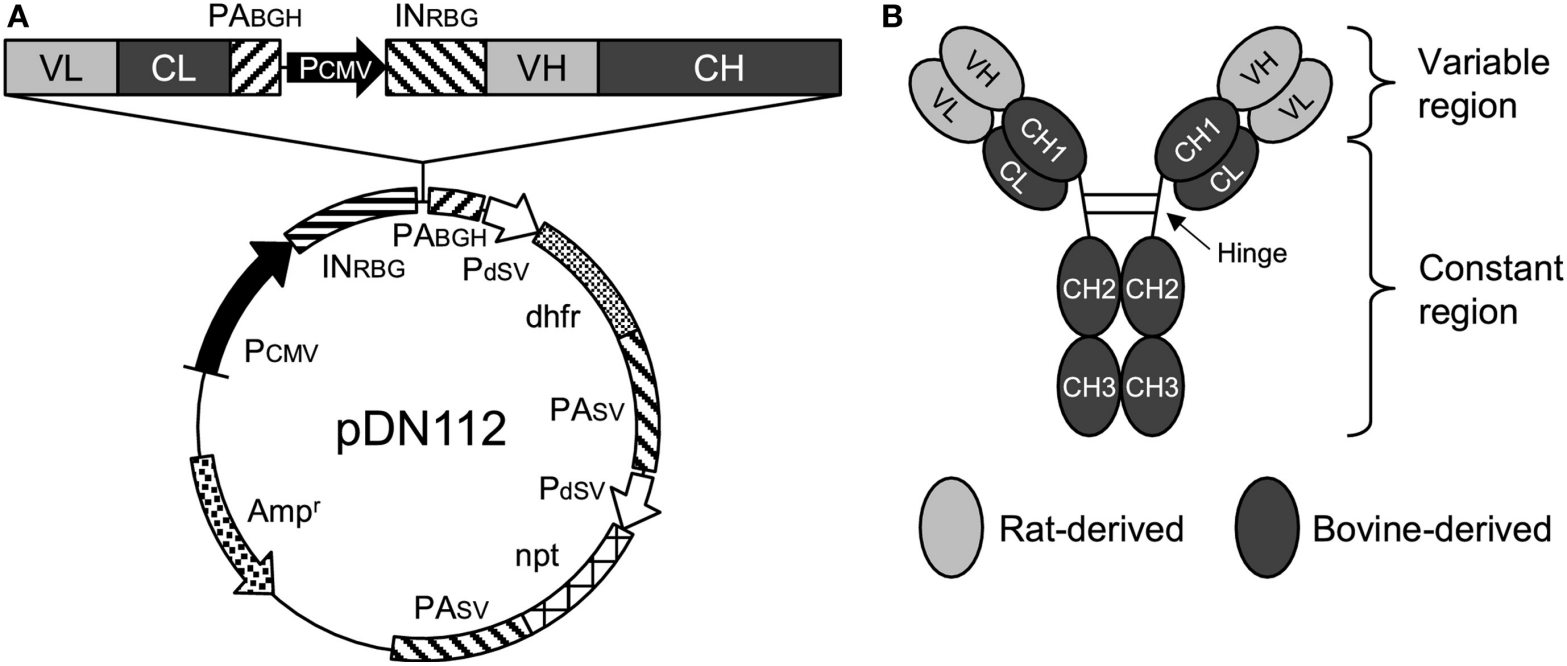
Anti-programmed death-1 chimeric antibody, Boch5D2. **(A)** Schematic structure of a plasmid vector encoding Boch5D2 (pDN112-Boch5D2 IgG_1_ ADCC−). A light chain consists of a variable region (VL) and a constant region (CL). A heavy chain consists of a variable region (VH) and a constant region (CH: CH1, hinge, CH2, and CH3). The pDN112 vector includes a neomycin-resistant gene (npt), and a dihydrofolate reductase gene (dhfr). **(B)** Schematic structure of Boch5D2. Boch5D2 is composed of two identical heavy chains and light chains, as with the normal IgG antibody.

The nucleotide sequences of the variable regions of the heavy and light chains of 5D2 were combined with the constant regions of bovine IgG_1_ (GenBank accession number X62916) and bovine Ig lambda (GenBank accession number X62917), respectively. For the preparation of Boch5D2 with IgG1 triggering reduced Fc-mediated effector functions (Boch5D2 IgG_1_ ADCC−), amino acid mutations were introduced into the binding sites for FcγRs of bovine IgG_1_ CH2 domain (Figure S3A in Supplementary Material) (36–38). The designed sequences were modified according to the optimal codon usage of Chinese hamster, synthesized (Integrated DNA Technologies, Coralville, IA, USA), and cloned into a pDN112 expression vector (pDN11 with a modified multicloning site) (Figure 1A) (38, 39).

Stable high-producer cell lines expressing Boch5D2 were established with the use of the dihydrofolate reductase (dhfr)/methotrexate gene amplification system in dhfr-deficient (dhfr^−/−^) Chinese hamster ovary (CHO) DG44 cells. CHO DG44 cells were transfected with pDN112-Boch5D2 IgG_1_ ADCC− and selected in CD OptiCHO medium (Thermo Fisher Scientific) supplemented with 2 mM GlutaMAX-I (Thermo Fisher Scientific) and 800 µg/ml G418 sulfate (Enzo Life Sciences, Farmingdale, NY, USA). After 3 weeks, the cells were screened for the ability to produce Boch5D2 by dot blotting and enzyme-linked immunosorbent assay (ELISA) with horseradish peroxidase (HRP)-conjugated anti-bovine IgG Fc rabbit polyclonal antibody (Rockland Immunochemicals, Pottstown, PA, USA), as previously described (38). Single-cell cloning of the polyclonal cell lines obtained above was performed by limiting dilution and screened again as described above. Gene amplification of the single-cell clones was subsequently performed in CD OptiCHO medium (Thermo Fisher Scientific) containing 60 nM methotrexate (Enzo Life Sciences). Boch5D2 was produced by shaking cultivation of the established cell lines producing the highest amount of antibody in G418- and methotrexate-free CD OptiCHO medium (Thermo Fisher Scientific) at 37°C and 125 rpm with 5% CO_2_ for 14 days. Live and dead cells were counted with a Countess Automated Cell Counter (Thermo Fisher Scientific) on days 3, 7, 10, and 14. At the same time points, the concentration of Boch5D2 in the culture supernatant was determined by bovine IgG ELISA, as described above.

### Purification of Boch5D2

Purification of Boch5D2 from the culture supernatant was performed by affinity chromatography with an Ab-Capcher ExTra (ProteNova, Kagawa, Japan), and the buffer was exchanged with phosphate-buffered saline (PBS) by size exclusion chromatography using PD-10 Desalting Column (GE Healthcare, Buckinghamshire, England, UK). The concentration of Boch5D2 was measured by ultraviolet (UV) absorbance at 280 nm with a NanoDrop 8000 Spectrophotometer (Thermo Fisher Scientific). The purity of Boch5D2 was confirmed by sodium dodecyl sulfate-polyacrylamide gel electrophoresis (SDS-PAGE) in reducing or non-reducing condition using 10% polyacrylamide gel and 2× Laemmli Sample Buffer (Bio-Rad, Hercules, CA, USA). Precision Plus Protein All Blue Standard (Bio-Rad) was used as a molecular-weight size marker, and the proteins were visualized with Quick-CBB (Wako Pure Chemical Industries, Osaka, Japan). The purity of Boch5D2 was evaluated by densitometry with CS Analyzer Software version 3.0 (Atto, Tokyo, Japan) and was routinely >90%.

### Binding Assay of Boch5D2 to Membrane-Bound Bovine PD-1

To confirm the binding activity of Boch5D2 to membrane-bound bovine PD-1, flow cytometric analyses were performed using myc-tagged bovine PD-1-expressing CHO DG44 cells (BoPD-1-myc cells) (25). Briefly, BoPD-1-myc cells were incubated with 5D2 (25) or Boch5D2 at room temperature for 30 min. Rat IgG_2a_ (R35-95, BD Biosciences, San Jose, CA, USA) and bovine IgG_1_ antibodies (Bethyl Laboratories, Montgomery, TX, USA) were used as isotype controls. The cells were then washed with PBS and labeled with APC-conjugated anti-rat immunoglobulin antibody (Southern Biotech, Birmingham, AL, USA) or APC-conjugated anti-bovine IgG Fc goat antibody (Jackson ImmunoResearch, West Grove, PA, USA) at room temperature for 30 min. Finally, the cells were washed and analyzed immediately using FACS Verse (BD Biosciences) and FCS Express 4 (De Novo Software, Glendale, CA, USA). The primary antibodies used in this experiment are also shown in Table 1.

**Table 1:** Primary antibodies used in flow cytometric analyses of this study.

Target	Isotype	Clone	Source	Fluorochrome	Conjugation or labeling
**Binding assay of anti-programmed death-1 (PD-1) antibodies**					
PD-1	Bovine IgG_1_	Boch5D2	This study	Alexa Fluor 647	Alexa Fluor 647-conjugated anti-bovine IgG Fc antibody (Jackson ImmunoResearch)
Bovine IgG_1_ isotype control	Bovine IgG_1_	Poly	Bethyl	Alexa Fluor 647	Alexa Fluor 647-conjugated anti-bovine IgG Fc antibody (Jackson ImmunoResearch)
PD-1	Rat IgG_2a_	5D2	In house (25)	APC	APC-conjugated anti-rat Ig antibody (Southern Biotech)
Rat IgG_2a_ isotype control	Rat IgG_2a_	R35-95	BD Biosciences	APC	APC-conjugated anti-rat Ig antibody (Southern Biotech)

**Flow cytometric analysis of T-cell proliferation**					
CFSE	–	–	Sigma-Aldrich	CFSE	–
CD3	Mouse IgG_1_	MM1A	WSU Monoclonal Antibody Center	PE	Zenon R-PE Mouse IgG_1_ Labeling Kit (Thermo Fisher Scientific)
CD4	Mouse IgG_1_	CC30	Bio-Rad	Alexa Fluor 647	Zenon Alexa Fluor 647 Mouse IgG_1_ Labeling Kit (Thermo Fisher Scientific)
CD8	Mouse IgG_2a_	CC63	Bio-Rad	PerCp/Cy5.5	Lightning-Link PerCp/Cy5.5 Conjugation Kit (Innova Biosciences)
TCR1-N24 (δ chain)	Mouse IgG_2b_	GB21A	WSU Monoclonal Antibody Center	APC/Cy7	Lightning-Link APC/Cy7 Conjugation Kit (Innova Biosciences)
IgM	Mouse IgG_1_	IL-A30	Bio-Rad	PE/Cy7	Lightning-Link PE/Cy7 Conjugation Kit (Innova Biosciences)

*CFSE, carboxyfluorescein diacetate succinimidyl ester; PE, phycoerythrin; PerCp, peridinin–chlorophyll–protein complex; APC, allophycocyanin; Cy, cyanin*.

### Surface Plasmon Resonance (SPR) Analysis

To assess the binding affinity of 5D2 and Boch5D2 to bovine PD-1, SPR analysis was performed using the Biacore system (GE Healthcare) with polyhistidine-tagged bovine PD-1 protein (BoPD-1-His). For the preparation of BoPD-1-His, cDNA encoding the extracellular domain fragment of bovine PD-1 (GenBank accession number AB510901) with a signal sequence was amplified by PCR with gene-specific primers with a Kozak sequence, a C-terminal 6× histidine tag-encoding sequence, and restriction enzyme cleavage sites (Table S1 in Supplementary Material). The amplicon was then cloned into the multicloning site of pCXN2.1(+) (kindly provided by Dr. T. Yokomizo, Juntendo University, Japan) (40). A transient cell line expressing BoPD-1-His was established with the use of Expi293 Expression System (Thermo Fisher Scientific). Briefly, Expi293F cells were transfected with pCXN2.1(+)-BoPD-1-His with the use of ExpiFectamine (Thermo Fisher Scientific) and cultivated with shaking in Expi293 medium (Thermo Fisher Scientific) at 37°C and 125 rpm with 8% CO_2_ for 7 days. BoPD-1-His was purified from the culture supernatant with TALON Metal Affinity Resin (Clontech, Palo Alto, CA, USA), and the buffer was exchanged with PBS as described above. The purity of BoPD-1-His was confirmed by SDS-PAGE, and the concentration of BoPD-1-His was determined with a NanoDrop 8000 Spectrophotometer (Thermo Fisher Scientific) as described above.

Surface plasmon resonance measurement was performed on a Biacore X100 instrument (GE Healthcare) at 25°C. Purified BoPD-1-His was immobilized on a CM5 sensor chip (GE Healthcare) by an Amine Coupling Kit (GE Healthcare) following the manufacturer’s instructions to analyze 5D2 or Boch5D2 binding. HBS-EP+ (GE Healthcare) was used for both the running and the dilution buffers. Control run responses containing buffer only were subtracted to obtain specific binding responses. The kinetic constants of 5D2 and Boch5D2 were determined by fitting with the 1:1 kinetic binding model.

### Blockade Assay of PD-1/PD-L1 Binding

Bovine PD-1-bovine IgG Fc fusion protein (BoPD-1-Ig) was expressed in a previously established stable expression cell line, purified, and quantified as described previously (38). To confirm the ability of 5D2 and Boch5D2 to block PD-1/PD-L1 binding, biotinylated BoPD-1-Ig (5 µg/ml) was incubated with various concentrations (0.39–50 µg/ml) of 5D2 and Boch5D2 at 37°C for 30 min. The incubated BoPD-1-Ig proteins were then reacted with bovine PD-L1-EGFP-expressing CHO DG44 cells (BoPD-L1-EGFP cells) (38) at 37°C for 30 min. BoPD-1-Ig bound to BoPD-L1-EGFP cells was labeled with APC-conjugated streptavidin (BioLegend, San Diego, CA, USA) at room temperature for 30 min, washed with PBS, and analyzed immediately by FACS Verse (BD Biosciences). Rat IgG_2a_ (R35-95, BD Biosciences) and bovine IgG_1_ antibodies (Bethyl Laboratories) were used as isotype controls.

### Administration of Boch5D2 to Cattle

To confirm the effects of Boch5D2 in cattle (*in vivo*), a BLV-infected calf (animal number 15-6; Holstein, male, 173 kg, 4 months old) was administered 14 mg (0.08 mg/kg) of purified Boch5D2 intravenously. Peripheral blood was collected before inoculation and more than once a week after inoculation. This animal was inoculated with 1.4 × 10^8^ leukocytes infected with BLV (1.4 × 10^7^ copies of provirus) 8 weeks before antibody inoculation and developed the aleukemic stage of BLV infection. Infected leukocytes were isolated from the blood of a BLV-infected cow in the persistent lymphocytosis stage. These animals were kept in a biosafety level I animal facility at the Animal Research Center, Agricultural Research Department, Hokkaido Research Organization (Shintoku, Hokkaido, Japan). This animal experiment was approved by the Ethics Committee of the Animal Research Center, Agricultural Research Department, Hokkaido Research Organization.

### Detection of Boch5D2 in Serum of the Inoculated Cattle

To determine the kinetics of Boch5D2 in the serum of animal 15-6, anti-PD-1 antibody was detected by ELISA with BoPD-1-His protein. BoPD-1-His was diluted to 10 µg/ml in 0.05 M carbonate–bicarbonate buffer (Sigma-Aldrich, St. Louis, MO, USA) and coated onto Nunc MaxiSorp ELISA plates (Nunc, Roskilde, Denmark) at 4°C overnight. The plates were washed with Tris-buffered saline supplemented with 0.05% Tween20 (TBS-T) and incubated with TBS-T containing 1% skim milk at room temperature for 1 h. After washing with TBS-T, the serum samples of animal 15-6 were incubated in triplicate at room temperature for 1 h. The plates were washed again with TBS-T, and antibody binding to PD-1 was detected by HRP-conjugated anti-bovine IgG Fc rabbit polyclonal antibody (Rockland Immunochemicals) and TMB One Component Substrate (Bethyl Laboratories). The reported values are the means of triplicate samples.

### Cell Proliferation Assay

To investigate the effect of PD-1 blockade on the BLV-specific T-cell response, cell proliferation assays were performed. Peripheral blood mononuclear cells (PBMCs) were purified from the blood samples by density gradient centrifugation on Percoll (GE Healthcare), washed three times with PBS, and suspended in PBS. Isolated PBMCs were then labeled with carboxyfluorescein diacetate succinimidyl ester (CFSE) (Sigma-Aldrich) and cultured in triplicate with 2% heat-inactivated culture supernatant of BLV-infected fetal lamb kidney (FLK) cells (41) or BLV gp51 peptide mix [0.1 and 1 µg/ml of each peptide (25)] for 6 days. The heat-inactivated culture supernatant of BLV-uninfected FLK cells was used as a negative control antigen. All cell cultures were grown in 96-well round-bottomed plates (BD Biosciences) containing 1 × 10^6^ PBMCs in 250 µl RPMI 1640 medium (Sigma-Aldrich) supplemented with 10% heat-inactivated fetal bovine serum (Cansera International, Etobicoke, ON, Canada), 200 IU/ml of penicillin, 200 µg/ml of streptomycin, and 0.01% l-glutamine (Thermo Fisher Scientific) at 37°C with 5% CO_2_. After 6 days, the PBMCs were harvested and incubated in PBS containing 10% goat serum (Sigma-Aldrich) at room temperature for 15 min to prevent non-specific reactions. The cells were then stained with anti-CD3-PE (MM1A; Washington State University Monoclonal Antibody Center, Pullman, WA, USA), anti-CD4-Alexa Fluor 647 (CC30; Bio-Rad), anti-CD8-PerCp/Cy5.5 (CC63, Bio-Rad), anti-TCR1-N24-APC/Cy7 (anti-TCR δ chain; GB21A; Washington State University Monoclonal Antibody Center), and anti-IgM-PE/Cy7 antibodies (IL-A30; Bio-Rad) at 4°C for 30 min. MM1A and CC30 were pre-labeled with R-PE and Alexa Fluor 647 with Zenon Mouse IgG_1_ Labeling Kits (Thermo Fisher Scientific). CC63, GB21A, and IL-A30 were conjugated with PerCp/Cy5.5, APC/Cy7, and PE/Cy7, respectively, with Lightning-Link Conjugation Kits (Innova Biosciences, Cambridge, England, UK). The cells were then washed with PBS containing 1% bovine serum albumin (Sigma-Aldrich) and analyzed immediately by FACS Verse (BD Biosciences) and FCS Express 4 (De Novo Software).

### Quantification of BLV Proviral Load

To determine proviral loads in the Boch5D2-inoculated animal, BLV *tax* gene was measured by quantitative real-time PCR. Briefly, genomic DNA was extracted from 2 × 10^6^ PBMCs with a Wizard Genomic DNA Purification Kit (Promega). Amplification of the BLV *tax* gene was performed in a reaction mixture containing 5 µl of Cycleave PCR Reaction Mix (Takara Bio, Otsu, Japan), 0.5 µl of Probe/Primer Mix for BLV (Takara Bio), 1 µl of a DNA template, and 3.5 µl of RNase-Free Distilled Water (Takara Bio) with a LightCycler 480 system II (Roche Diagnostics, Mannheim, Germany). Serial dilution of BLV Positive Control (Takara Bio) was used to generate calibration curves to determine the copy number of the BLV *tax* gene. Each DNA sample was tested in triplicate, and the reported values are the mean numbers of copies per 50 ng of DNA. The concentration of DNA was measured by UV absorbance at 260 nm with a NanoDrop 8000 Spectrophotometer (Thermo Fisher Scientific).

### Statistical Analysis

Significant differences were identified by Welch’s *t*-test and repeated one-way analysis of variance, followed by Dunnett’s test. All statistical tests were performed with GraphPad Prism 6 (GraphPad Software, San Diego, CA, USA). Differences were considered statistically significant when *P* < 0.05.

## Results

### Treatment of Anti-PD-1 Rat mAb in BLV-Infected Cattle

To evaluate the therapeutic effects of PD-1 blockade *in vivo*, a BLV-infected cow was inoculated with anti-PD-1 rat mAb (5D2). The serum concentration of the inoculated 5D2 was high during the first week postinoculation and decreased from 11 days postinoculation (dpi) (Figure S1 in Supplementary Material). At 18 dpi, 5D2 was not detected in the serum (Figure S1 in Supplementary Material). Before inoculation, *ex vivo* culture of PBMCs resulted in low or no production of interferon-γ (IFN-γ) in response to BLV gp51 peptides (Figure S2A in Supplementary Material), indicating the functional exhaustion of gp51-specific T cells in this animal. After inoculation, however, gp51-specific IFN-γ production was slightly activated until 25 dpi (Figure S2A in Supplementary Material), suggesting that PD-1 blockade partially restores the effector function of BLV-specific T cells *in vivo*. Despite enhancement of the BLV-specific T-cell response, the proviral load of BLV did not change in the animal throughout the experiment (Figure S2B in Supplementary Material).

### Establishment of the Anti-Bovine PD-1 Rat–Bovine chAb, Boch5D2

We hypothesized that treatment with anti-PD-1 rat mAb 5D2 could not induce antiviral effects because 5D2 was rapidly eliminated as a heterologous protein in cattle. To obtain a blocking antibody that was more stable *in vivo*, 5D2 was then engineered into an anti-bovine PD-1 rat–bovine chimeric monoclonal antibody, Boch5D2 (Figure 1B). Additionally, Boch5D2 is desired to induce no Fc-mediated effector functions, such as antibody-dependent cell-mediated cytotoxicity (ADCC). Thus, we introduced amino acid mutations into possible binding sites for FcγRs of a CH2 domain of bovine IgG_1_ (Figure S3A in Supplementary Material) according to the confirmed mutations on human IgG_1_ (34, 35). Homology modeling predicted the structure of the constant region of bovine IgG_1_ and revealed that the target residues for mutation were located in the upper CH2 domain near the hinge region (Figure S3B in Supplementary Material). These residues were estimated to form the binding sites for FcγRs like human IgG_1_ (37). We then developed Boch5D2 IgG_1_ variants with wild type (IgG_1_ WT) and mutated constant regions (IgG_1_ ADCC−) in Expi293 Expression System (Figure S4A in Supplementary Material). Soluble bovine FcγR proteins were also prepared in Expi293 Expression System (Figure S4B in Supplementary Material) and tested for the bindings of Boch5D2 IgG_1_ WT and ADCC− (Figure S5 in Supplementary Material). Boch5D2 IgG_1_ WT bound strongly to bovine FcγRI, weakly to bovine FcγRII, but not to bovine FcγRIII and Fcγ2R (Figure S5 in Supplementary Material). The mutations introduced into the CH2 domain of Boch5D2 IgG_1_ ADCC− were effective to diminish the interactions with bovine FcγRI and FcγRII (Figures S5A,B in Supplementary Material). Thus, Boch5D2 IgG_1_ ADCC− is expected to induce no effector functions *via* FcγRs and is a suitable form as a blocking antibody targeting PD-1.

### Establishment of Stable High-Producer Cell Lines Expressing Boch5D2

**Figure 2:**
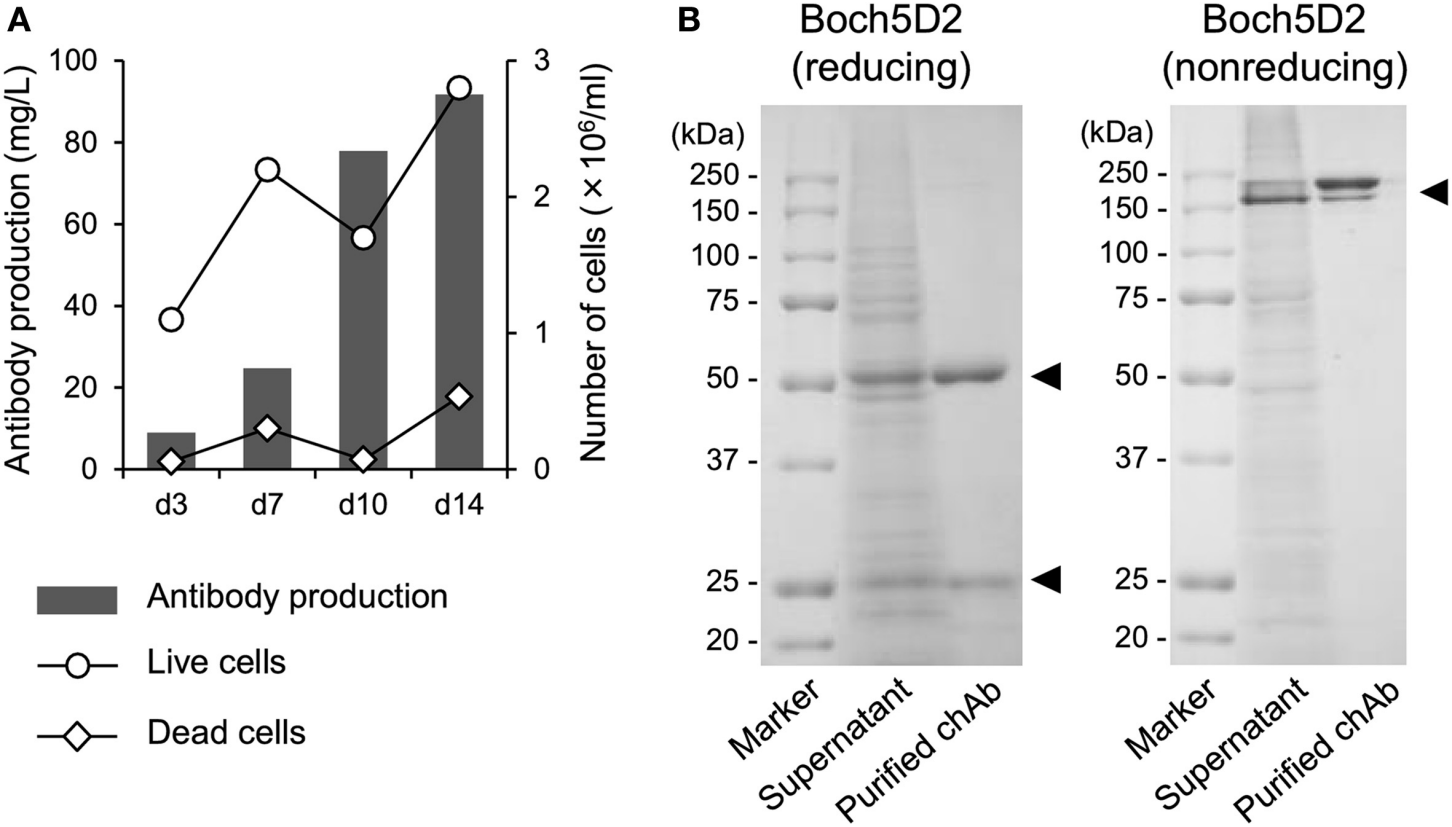
Production and purification of Boch5D2 in CHO DG44 cells. **(A)** Expression of Boch5D2. Boch5D2 was expressed stably in CHO DG44 cells in 30 ml of shaking culture. The numbers of live and dead cells (right axis: white circle and diamond) and antibody production (left axis: gray bar) were measured at 3- to 4-day intervals. **(B)** Purification of Boch5D2. Boch5D2 was purified from supernatants of shaking cultures. Purified protein was confirmed by reducing and non-reducing sodium dodecyl sulfate-polyacrylamide gel electrophoresis.

To obtain large amount of Boch5D2 (IgG_1_ ADCC−) for further characterization *in vitro* and *in vivo*, Boch5D2 (IgG_1_ ADCC−) was stably expressed and produced with the use of the CHO DG44 cell expression system. The highest-producing cell line in CHO DG44 cells stably produced 91.7 mg/l of Boch5D2 after 14 days of shaking culture (Figure 2A). Boch5D2 was successfully purified from supernatants with the use of Protein A resin (Figure 2B). As expected, the heavy and light chains of Boch5D2 were detected at approximately 50 and 25 kDa, respectively (Figure 2B). Thus, Boch5D2 has been successfully established and produced with the use of mammalian expression system.

### Reactivities of Boch5D2 to Membrane-Bound Bovine PD-1

**Figure 3:**
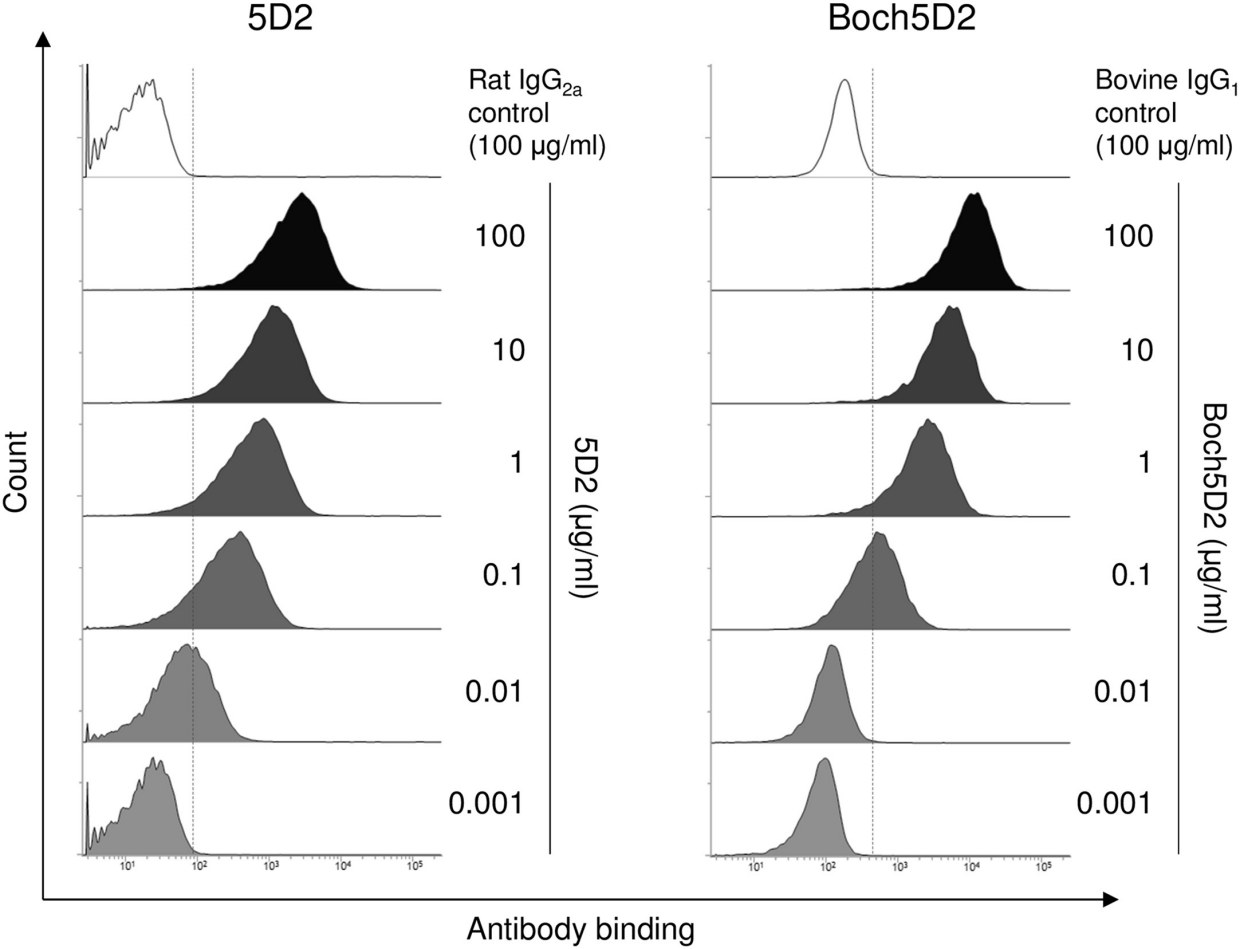
Reactivity of Boch5D2 with BoPD-1-myc cells. Flow cytometric analyses using Boch5D2. BoPD-1-myc cells were stained with 5D2 and Boch5D2 in serial dilutions (from 100 µg/ml to 1 ng/ml).

The binding ability of anti-PD-1 chAb was determined by flow cytometric analysis with membrane-bound PD-1-expressing cells. Flow cytometric analysis showed that Boch5D2 bound to more than 95% of PD-1-expressing cells at 1–100 µg/ml and to 62% at 0.1 µg/ml (Figure 3), representing a binding ability similar to that of 5D2. The Boch5D2 produced in this study is capable of detecting its target with reactivity similar to that of the original rat mAb.

### Binding Affinity of Boch5D2 to Bovine PD-1 Protein

To confirm the binding affinity of Boch5D2 to PD-1 protein, BoPD-1-His was prepared in Expi293 Expression System (Figure S6 in Supplementary Material), and SPR analysis was performed with the use of a Biacore instrument. 5D2 and Boch5D2 bound to BoPD-1-His protein successfully. As shown in Table 2, 5D2 and Boch5D2 showed significantly similar affinities for PD-1, with *K*_D_ values of 0.12 ± 0.04 and 0.10 ± 0.06 nM, respectively. Therefore, the chimerization of anti-PD-1 antibody does not change its binding affinity to bovine PD-1 protein.

**Table 2:** Binding affinity of 5D2 and Boch5D2 to BoPD-1-His protein.

Antibody	*k*_a_ (1/Ms)	*k*_d_ (1/s)	*K*_D_ (M)^a^
5D2	1.84 × 10^4^ ± 0.27	2.15 × 10^−4^ ± 0.44	1.22 × 10^−8^ ± 0.39
Boch5D2	2.07 × 10^4^ ± 0.06	2.16 × 10^−4^ ± 1.12	1.05 × 10^−8^ ± 0.58

*^a^The *K*_D_ values of 5D2 and Boch5D2 are not significantly different (*P* > 0.05)*.

### Blockade of PD-1/PD-L1 Binding by Boch5D2

**Figure 4:**
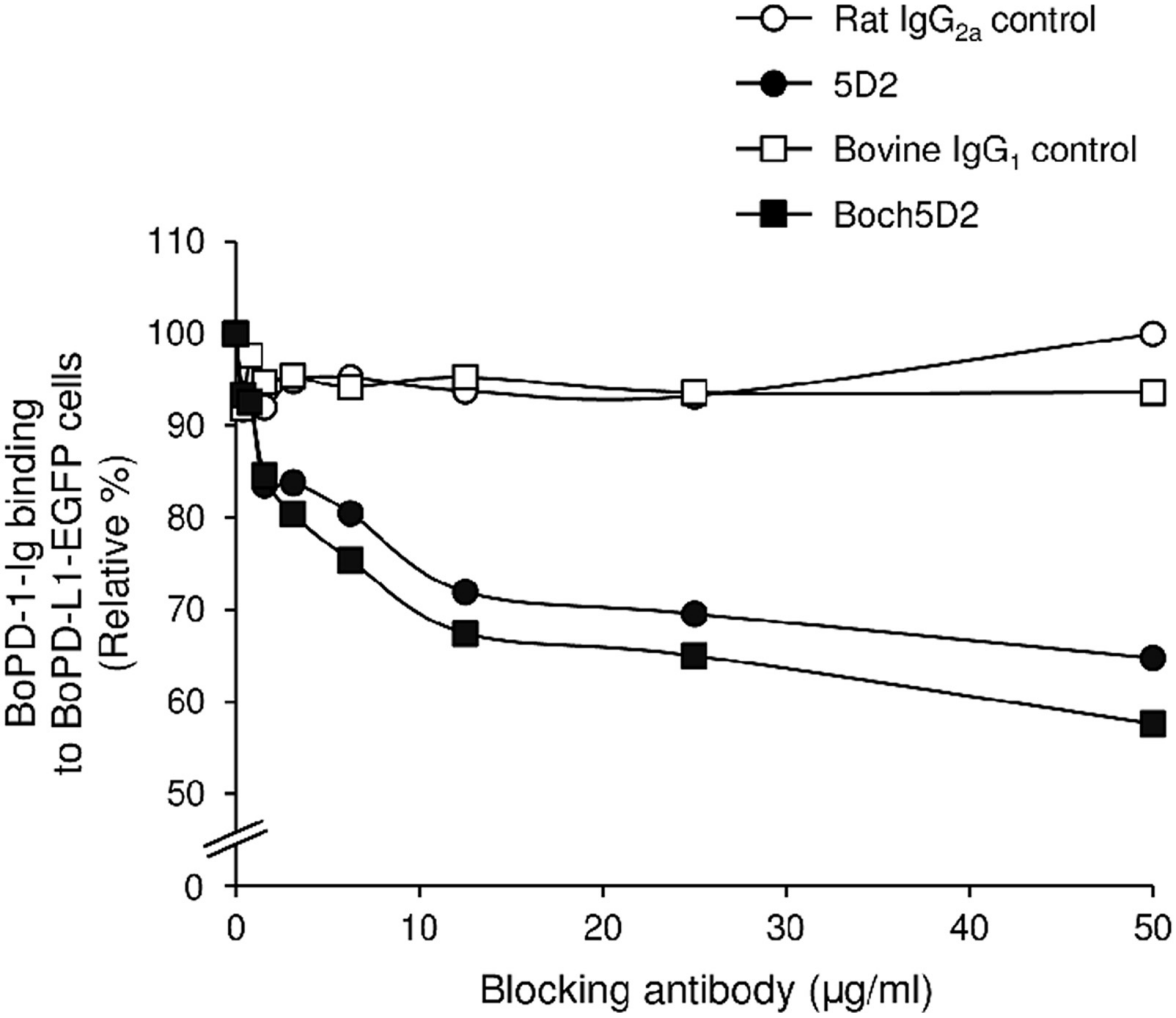
Blockade of programmed death-1 (PD-1)/PD-ligand 1 (PD-L1) binding by Boch5D2. BoPD-1-Ig was preincubated with 5D2 and Boch5D2 and then reacted with BoPD-L1-EGFP cells. BoPD-1-Ig bindings were evaluated by flow cytometry. Each curve represents relative binding of BoPD-1-Ig preincubated with 5D2 and Boch5D2 compared to no-antibody control. Rat IgG_2a_ (for 5D2) and bovine IgG_1_ (for Boch5D2) were used as negative controls.

To analyze the blocking activity of Boch5D2, the inhibitory efficacy of chAb in PD-1-Ig binding to PD-L1-expressing cells was evaluated in cell-based experiments using flow cytometry. Preincubation of PD-1-Ig with either 5D2 or Boch5D2 inhibited PD-1/PD-L1 binding in a dose-dependent manner (Figure 4). In preincubations using 50 µg/ml of the antibodies, such as Boch5D2 and 5D2, inhibited 42.4 and 35.2% of PD-1/PD-L1 binding, respectively (Figure 4). Thus, the blocking activity of Boch5D2 is similar to that of the original rat mAb 5D2.

### Reactivation of T-Cell Functions by Treatment with Boch5D2 in BLV-Infected Cattle

**Figure 5:**
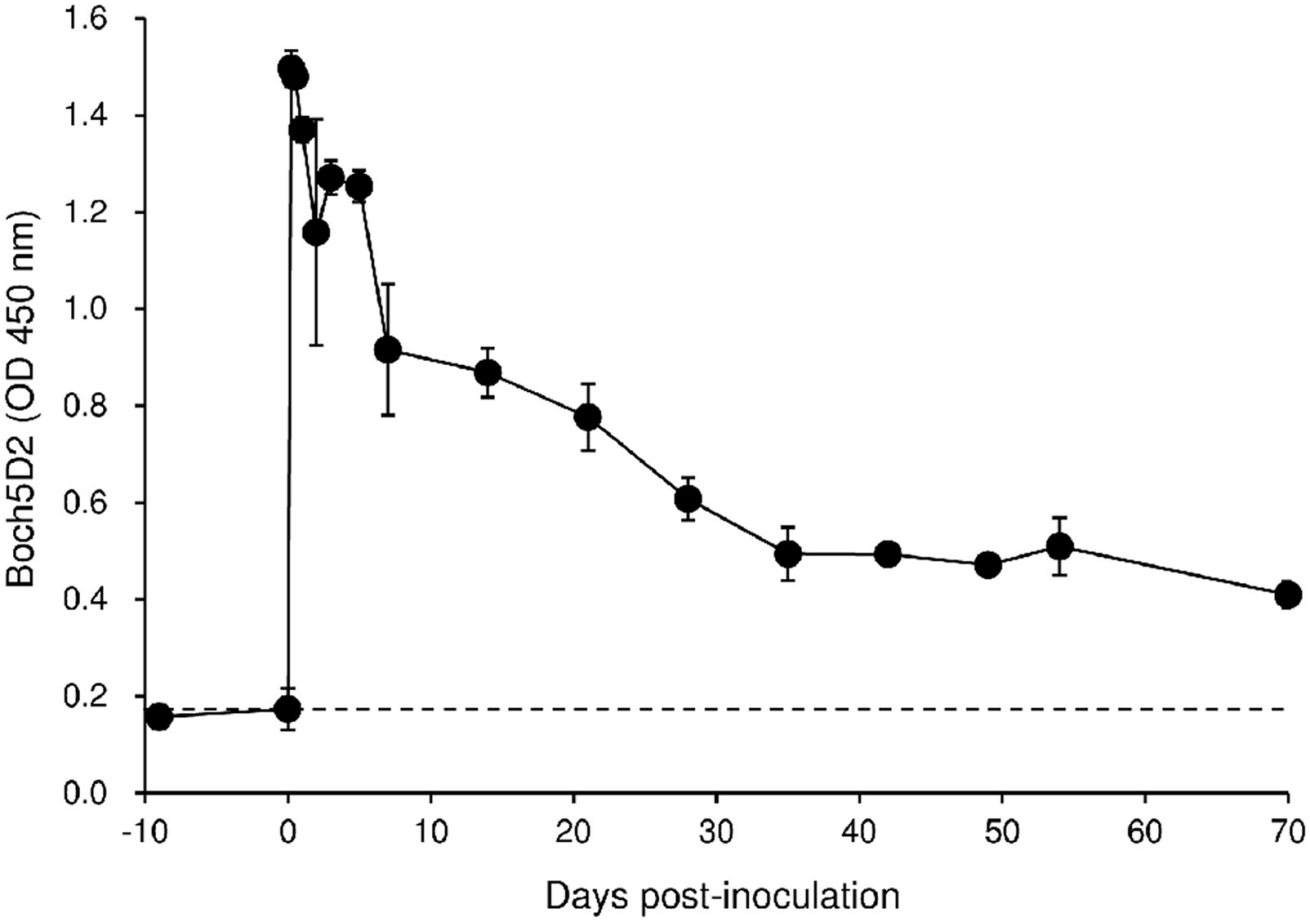
Kinetics of Boch5D2 in serum of the inoculated calf. A bovine leukemia virus-infected calf (*n* = 1) was inoculated with Boch5D2 (0.08 mg/kg). The serum concentration of Boch5D2 was determined by enzyme-linked immunosorbent assay precoated with BoPD-1-His protein. Each dot represents the mean of three independent experiments.

**Figure 6:**
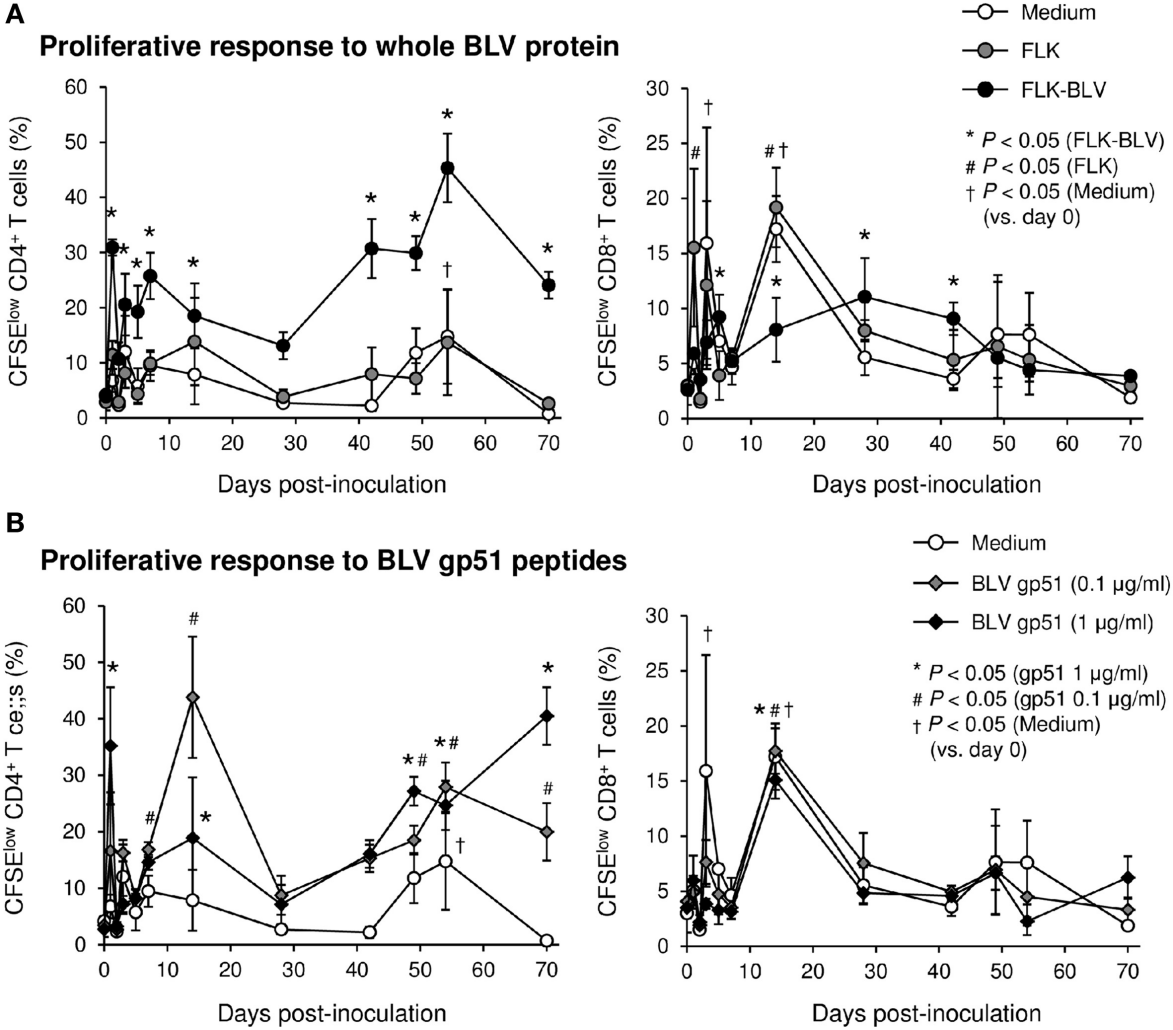
Effect on proliferation of bovine leukemia virus (BLV)-specific T cells of the administration of Boch5D2. T-cell proliferation specific for BLV antigen stimulation. Carboxyfluorescein diacetate succinimidyl ester (CFSE)-labeled peripheral blood mononuclear cells were cultured in triplicate with fetal lamb kidney (FLK)-BLV antigen, control FLK antigen **(A)**, or gp51 peptides (0.1 and 1 µg/ml) **(B)** for 6 days. The percentage of CFSE^low^ cells in CD4^+^ and CD8^+^γδTCR^−^ T cells was measured by flow cytometry. CFSE^low^ cells represent cells proliferated during cultivation. Each dot represents the mean of three independent experiments. Significant differences were determined by Dunnett’s multiple-comparison test across the time points. ^*,#,†^*P* < 0.05 versus 0 dpi in each stimulation.

To evaluate the therapeutic effects of Boch5D2 *in vivo*, a BLV-infected calf was inoculated with anti-PD-1 chAb (Boch5D2). The serum concentration of the inoculated Boch5D2 was relatively high during the first week postinoculation (Figure 5), showing a trend similar to that in a 5D2-inoculated animal (Figure S1 in Supplementary Material). Unlike the concentration of inoculated 5D2, the concentration of Boch5D2 decreased slowly, and it was still detectable in the serum at 70 dpi when this animal experiment was terminated (Figure 5). The BLV-specific proliferation of PBMCs was analyzed at 1- or 2-day intervals during the first week after inoculation. Activation of CD4^+^ T-cell proliferation stimulated by FLK-BLV antigen was significantly higher from 1 dpi than at 0 dpi (Figure 6A). In contrast, CD8^+^ T-cell proliferation was activated in response not only to FLK-BLV antigen but also to controls (FLK control antigen and medium) after Boch5D2 inoculation (Figure 6A). Furthermore, a proliferation assay with BLV gp51 peptides showed that CD4^+^ T-cell proliferation in response to the peptide antigens was significantly enhanced after inoculation, but that of CD8^+^ T cells was not (Figure 6B). Surprisingly, the enhanced proliferation of BLV-specific CD4^+^ T cells was not impaired at 70 dpi (Figures 6A,B). A single treatment with Boch5D2 activated the effector function of T cells, including BLV-specific CD4^+^ T cells, in a BLV-infected animal in the long term.

### Reduction of Proviral Load by the Treatment with Boch5D2 in BLV-Infected Cattle

**Figure 7:**
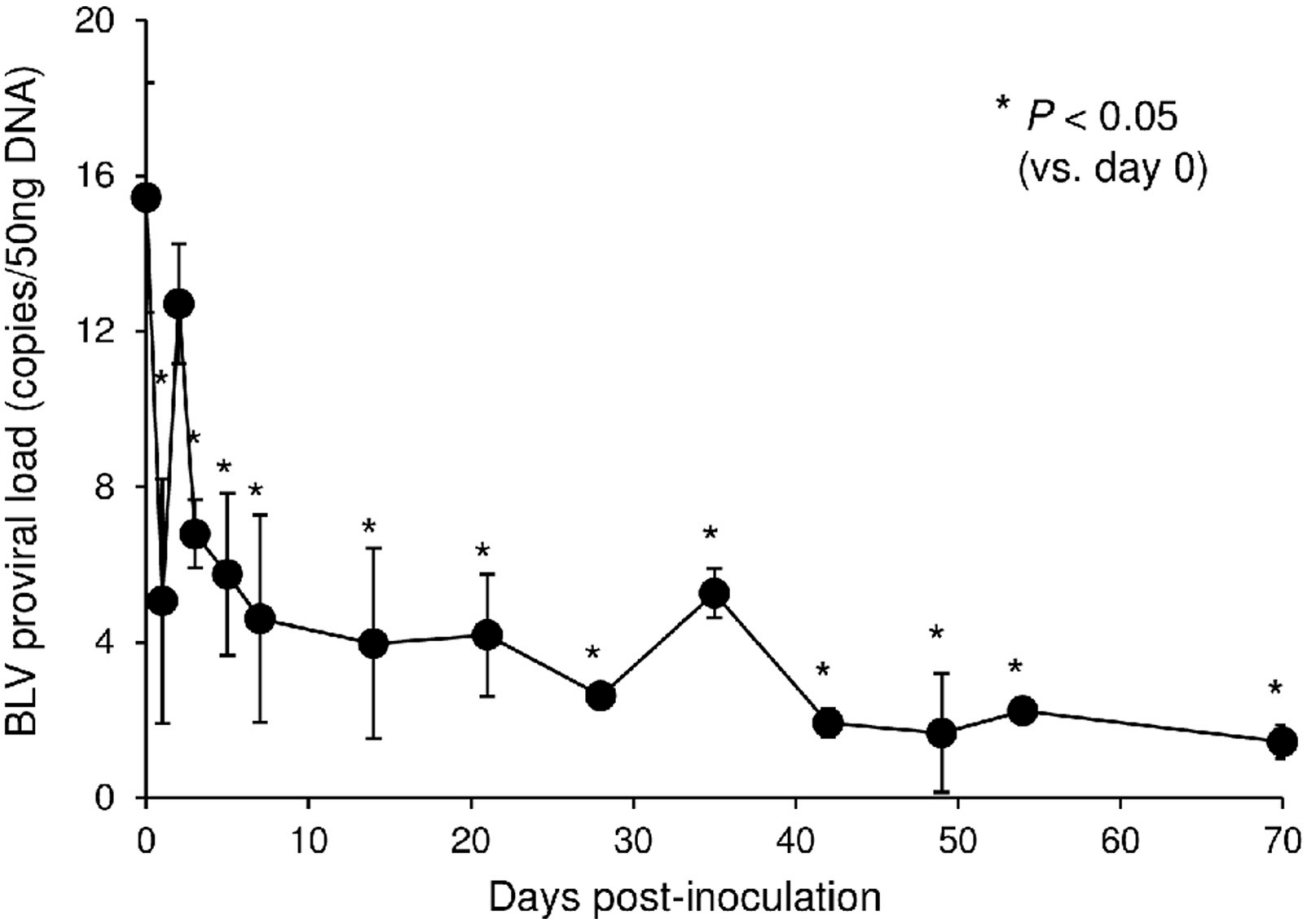
Effect on proviral loads of bovine leukemia virus (BLV) in a calf-administered Boch5D2. Provirus copy number per 50 ng DNA of peripheral blood mononuclear cells (PBMCs) from an inoculated calf. Proviral loads of BLV were quantified in PBMCs at each time point by real-time genomic polymerase chain reaction targeting the BLV *tax* gene. Each dot represents the mean of three independent experiments. Significant differences were determined by Dunnett’s multiple-comparison test across the time points. *P < 0.05 versus 0 dpi.

In this tested animal, the BLV proviral load in PBMCs was 15.4 copies/50 ng DNA before inoculation (at 0 dpi) (Figure 7). This animal remained in the aleukemic stage of BLV infection throughout the experimental period, such that the proviral load was low but detectable. After inoculation with Boch5D2, the proviral load decreased significantly after 1 dpi (5.0 copies/50 ng DNA) (Figure 7), consistent with the enhancement of the BLV-specific T-cell response (Figure 6). The proviral load did not increase after 3 dpi; at 70 dpi, it was 1.4 copies/50 ng DNA, which was 10.7-fold lower than at 0 dpi (Figure 7). Consequently, *in vivo* blockade of PD-1/PD-L1 resulted in a prolonged decrease in BLV-infected lymphocytes in the BLV-infected animal.

## Discussion

In this study, anti-bovine PD-1 chAb Boch5D2 was established in mammalian expression systems and exhibited equivalent *in vitro* activities to the original mAb 5D2 in terms of the binding affinity to bovine PD-1 protein and the blocking activity on bovine PD-1/PD-L1 interaction. Furthermore, the bovinized chimerization of anti-PD-1 mAb improved its *in vivo* stability presumably due to reduced immune response against constant regions of the inoculated antibodies, which is a strong advantage of this strategy. The chimerization technology will be useful to the development of therapeutic antibodies as well as cell type-specific antibodies for *in vivo* depletion experiments.

We administrated Boch5D2 to a small calf (173 kg) at a low dose (0.08 mg/kg) as a pilot trial, but administration of the chAb to adult cattle at higher dose requires gram-scale quantities of the chAb. For example, 0.5 g of chAb is required for administration to a 500-kg adult cow at a dose of 1 mg/kg. The current capacity of chAb production in CHO DG44 cell lines is still poor (about 0.1 g/l) and could be improved by further gene amplification using methotrexate at higher concentrations. In addition, large-scale culture using larger culture flasks and bioreactors is helpful for producing large amounts of chAbs (42). In recent studies, higher peak cell concentrations and product titers of up to 5 g/l have been routinely achieved in CHO DG44 cells, as production processes have steadily improved through advances in optimization of selection processes, basal media, and feed supplements (42–44). Thus, optimization of basal media and feed supplements will be among the next strategies for improving the established high-producing cell lines.

Although binding specificity and affinity to antigen are central for functional activity of antibody drug, the adequate choice of the constant region of the heavy chain is also essential to obtain expected effects of antibody treatment. IgG subclass inducing no Fc-mediated effector functions, such as ADCC, complement-dependent cytotoxicity (CDC), and antibody-dependent cell-mediated phagocytosis (ADCP), is considered to be suitable for the blocking antibody targeting PD-1 in human medicine (45, 46). Anti-human PD-1 antibodies launched for cancer therapy (nivolumab and pembrolizumab) are composed of human IgG_4_ heavy chain (46), which does not trigger ADCC and CDC functions *via* its Fc region (45).

Three IgG subclasses, IgG_1_, IgG_2_, and IgG_3_, have been described in cattle (47–49), and cattle do not produce functional IgG_4_ because bovine *IGHC4* gene encoding bovine IgG_4_ heavy chain exists as a pseudogene on the genome (50). In earlier studies, bovine IgG_1_ and IgG_2_ are clarified to mediate ADCC and ADCP (51, 52). Bovine IgG_3_ is characterized by a longer hinge sequence, which is presumably effective in the cross-linking with FcγRs and complement, and triggers active Fc-mediated effector functions like human IgG_3_ (45, 49). Thus, intact constant regions of bovine IgG subclasses may not be applied for effective blocking antibody. This study indicates that Boch5D2 IgG_1_ ADCC− does not interact with FcγRI and FcγRII, but Boch5D2 IgG_1_ WT does. The mutated bovine IgG_1_ may not trigger ADCC and ADCP and is a suitable form for blocking antibody. Further studies are needed to investigate ADCC, ADCP, and CDC functions of the Boch5D2 IgG_1_ variants and confirm the efficacy of the amino acid mutations in detail.

In the calf-administered Boch5D2, PD-1 blockade remarkably activated BLV-specific CD4^+^ T-cell proliferation. In contrast, the proliferation of BLV-specific CD8^+^ T-cells was limited before and after the inoculation. This result is consistent with the previous observation that CD4^+^ T cells express more PD-1 than do CD8^+^ T cells in BLV-infected cattle (25). Certainly, most of the PD-1^+^ cells were found among CD4^+^ T cells, with PD-1^+^CD8^+^ T cells representing a minority of the PD-1^+^ cells in this animal before Boch5D2 inoculation (data not shown). In addition, exogenous antigen is mainly recognized by CD4^+^ T cells *via* the MHC II pathways in proliferation assays. CD8^+^ T cells are also stimulated by exogenous antigens by cross-presentation *via* MHC I, but this stimulation is usually insufficient. Thus, BLV antigen-bearing antigen-presenting cells are required for detecting BLV-specific CD8^+^ T-cell responses in the assays. Most importantly, Boch5D2 treatment significantly decreased the BLV proviral load in the calf. This effect was presumably caused by the activation of BLV-specific CD4^+^ T cells. These data suggest that Boch5D2 may prevent disease progression in BLV infection through reduction of the viral load.

A hypothesis arises from the administration of anti-PD-1 rat mAb 5D2. In the BLV-infected cow inoculated with 5D2, PD-1 blockade unexpectedly restored the IFN-γ response specific for BLV gp51 antigens up to 25 dpi. Nevertheless, the BLV proviral load was not decreased in this animal. These results indicate that not only IFN-γ response but also other T-cell functions, such as proliferative activity, cytotoxic activity, and production of other T-cell cytokines (such as tumor necrosis factor-α and interleukin-2) and effector molecules (such as perforin and granzyme) may be involved in the control of BLV-infected cells *in vivo*.

The PD-1/PD-L1 pathway is expected to be a potential target for reinvigorating the function of exhausted T cells. A number of researchers and pharmaceutical companies have investigated antibody treatments that block the PD-1/PD-L1 pathway in humans, and anti-PD-1 and anti-PD-L1 antibodies have been approved for various human cancers or evaluated in clinical trials (7–11, 46). In the field of veterinary medicine, the PD-1/PD-L1 pathway has also attracted much attention for its potential as a novel target for cancer immunotherapy in companion animals (53–55). We recently established anti-bovine PD-L1 rat–bovine chAb as another candidate agent for blocking the PD-1/PD-L1 pathway (56). Administration of anti-PD-L1 chAb to a BLV-infected calf enhanced BLV-specific CD4^+^ T-cell proliferation and reduced BLV proviral load *in vivo*, which is consistent with the results in the current study. Thus, targeting the PD-1/PD-L1 pathway is a significant strategy for the regulation of T-cell response to pathogens in cattle.

The current work is a pilot study with a primary aim to determine the effects of Boch5D2 treatment. Although Boch5D2 treatment induced the immunomodulatory and antiviral effects in the initial trial, this result requires confirmation in a clinical trial with a large number of BLV-infected cattle from different herds or farms. Furthermore, we have revealed that T-cell exhaustion mediated by PD-1/PD-L1 presumably facilitates persistent infection and disease progression in BLV infection as well as bovine paratuberculosis, anaplasmosis, and mycoplasmosis (25–29). Clinical trials in cattle with paratuberculosis and anaplasmosis would determine whether Boch5D2 can be applied for a broad-spectrum immunotherapy against chronic infections of cattle. Additionally, expression and functional analyses are required in other infectious diseases of cattle, such as tuberculosis, chronic mastitis, theileriosis, and babesiosis, to expand the potential applications targeting the PD-1/PD-L1 pathway.

## Ethics Statement

This study was carried out in accordance with the recommendations of Guide for the Care and Use of Agricultural Animals in Research and Teaching, Federation of Animal Science Societies. The protocol was approved by the Ethics Committee of the Animal Research Center, Agricultural Research Department, Hokkaido Research Organization (Shintoku, Hokkaido, Japan) and by the Ethics Committee of Graduate School of Veterinary Medicine, Hokkaido University (Sapporo, Hokkaido, Japan).

## Author Contributions

SK, YS, SM, and KO were responsible for the conception and design of the study. TO, SK, AN, NM, RI, SG, JK, and SO performed the experiments. TO, SK, CN, JK, YK, YS, SM, and KO analyzed the data. CN, JK, SO, YK, and YS provided intellectual input, laboratory materials, reagents, and/or analytic tools. TO wrote the manuscript. SK, YK, YS, SM, and KO contributed to the revision of the manuscript. All the authors reviewed and approved the final manuscript.

## Conflict of Interest Statement

SK, KO, SM, TO, AN, NM, YS, and CN have a patent pending for materials and techniques described in this paper (Japanese patent, application number 2016-159090).
