# Supplementary material for: Anti-Bovine Programmed Death-1 Rat–Bovine Chimeric Antibody for Immunotherapy of Bovine Leukemia Virus Infection in Cattle
Source: Front Immunol. 2017 Jun 7;8:650. doi: 10.3389/fimmu.2017.00650 (PMC5461298; doi:10.3389/fimmu.2017.00650)
Supplement: Supplementary file 1 [file Presentation_1.pdf]

Supplementary Material

Anti-Bovine Programmed Death-1 Rat–Bovine Chimeric Antibody for Immunotherapy of Bovine Leukemia Virus Infection in Cattle

Tomohiro Okagawa, Satoru Konnai*, Asami Nishimori, Naoya Maekawa, Ryoyo Ikebuchi, Shinya Goto, Chie Nakajima, Junko Kohara, Satoshi Ogasawara, Yukinari Kato, Yasuhiko Suzuki, Shiro Murata, Kazuhiko Ohashi

*Correspondence: Satoru Konnai, konnai@vetmed.hokudai.ac.jp

Materials and methods

Administration of Anti-Bovine PD-1 Rat mAb in Cattle

To determine the immunomodulatory and antiviral effects of anti-bovine programmed death-1 (PD-1) blocking monoclonal antibody (mAb) in cattle, a bovine leukemia virus (BLV)-infected cow (animal ID HU-LOV; Holstein, female, 347 kg, 18 months old) was administrated 20 mg (0.06 mg/kg) of purified anti-bovine PD-1 rat mAb (5D2) (25) intravenously. This animal was naturally infected with BLV and developed aleukemic stage of the infection throughout the experiment. Peripheral blood was collected before inoculation and more than once a week after inoculation. The cow was kept in an animal facility at the Graduate School of Veterinary Medicine, Hokkaido University (Sapporo, Hokkaido, Japan). This animal experiment was approved by the Ethics Committee of Graduate School of Veterinary Medicine, Hokkaido University.

Detection of 5D2 in Serum of the Inoculated Cattle

To determine the kinetics of 5D2 in serum of animal HU-LOV, anti-PD-1 antibody was detected by ELISA with BoPD-1-His protein (10 μg/ml). BoPD-1-His was diluted to 10 μg/ml in 0.05 M carbonate-bicarbonate buffer (Sigma-Aldrich, St. Louis, MO, USA) and coated onto Nunc MaxiSorp ELISA plates (Nunc, Roskilde, Denmark) at 4°C overnight. The plate was washed with Tris-buffered saline supplemented with 0.05% Tween20 (TBS-T) and incubated with TBS-T containing 1% skim milk at room temperature for 1 h. After washing with TBS-T, the serum samples of animal HU-LOV were incubated in triplicate at room temperature for 1 h. The plates were washed again with TBS-T, and antibody binding to PD-1 was detected by horseradish peroxidase (HRP)-conjugated anti-

rat IgG goat polyclonal antibody (MP Biomedicals, Irvine, CA, USA) and TMB One Component Substrate (Bethyl Laboratories, Montgomery, TX, USA). The reported values are the means of triplicate samples.

Effector T-cell Response against BLV Antigen

To examine T-cell response to BLV antigen, freshly isolated peripheral blood mononuclear cells (PBMCs) were cultured with a mixture of BLV gp51 peptide mix (5 μg/ml of each peptide) (25) and control peptide (Flag peptide; 5 μg/ml) for 24 h. The culture supernatant was harvested and interferon-γ (IFN-γ) was determined by ELISA for bovine IFN-γ (Mabtech, Nacka Strand, Sweden) according to the manufacturer's protocol. Results were calculated based on a standard curve ranging from 7.8 to 500 pg/ml. The reported values are the means of duplicate samples.

Quantification of BLV Proviral Load

To determine proviral loads in the 5D2-inoculated animal, the BLV *tax* gene was measured by quantitative real-time polymerase chain reaction (PCR) with a Wizard Genomic DNA Purification Kit (Promega, Madison, WI, USA), Cycleave PCR BLV detection kit (Takara Bio, Otsu, Japan), and a LightCycler 480 system II (Roche Diagnostics, Mannheim, Germany), as described in the Materials and Methods.

***In silico* prediction of binding sites of Fcγ receptor on bovine IgG1**

To estimate binding sites of Fcγ receptor (FcγR) on bovine IgG_1, alignment analysis was conducted with the MEGA 6.0 software (57). Amino acid sequences of the constant

regions of bovine and human IgG1 (*IGHG1*; GenBank accession numbers CAA44699 and AAC82527, respectively) were aligned with the Clustal W program (58).

The structure of the constant region of bovine IgG_1 was predicted by automated homology modeling using SWISS-MODEL (59) from the amino acid sequence (GenBank accession number CAA44699). The crystal structure of human IgG_1 (PDB ID 1HZH) (60) was used as a template structure. The predicted structure was visualized in the Swiss PDB Viewer software (61).

Expression and purification of Boch5D2 IgG_1 variants and soluble bovine FcγRs

For the preparation of Boch5D2 with a wild-type constant region of bovine IgG_1 (Boch5D2 IgG_1 WT), cDNA encoding the constant region of bovine IgG_1 (GenBank accession number X62916) was amplified by PCR with gene-specific primers including restriction enzyme cleavage sites (Table S1). The amplicon was combined with the nucleotide sequence of the variable region of the heavy chain of 5D2 and cloned into a pDC6 expression vector (62) with the gene cassette encoding the chimeric light chain of Boch5D2.

For the preparation of polyhistidine (His)-tagged bovine FcγRI, FcγRII, FcγRIII, and Fcγ2R proteins (BoFcγRI-His, BoFcγRII-His, BoFcγRIII-His, and BoFcγ2R-His), cDNAs encoding the extracellular domain fragments of bovine FcγRI, FcγRII, FcγRIII, and Fcγ2R (GenBank accession numbers NM_174538, NM_174539, NM_001077402, and NM_001001138, respectively) with signal sequences were amplified by PCR with gene-specific primers with a Kozak sequence, a C-terminal 6× histidine tag-encoding sequence, and restriction enzyme cleavage sites (Supplementary Table 1). The amplicons

were then cloned into the multicloning site of pCXN2.1(+) (kindly provided by Dr. T. Yokomizo, Juntendo University, Japan) (40).

Transient cell lines expressing the Boch5D2 IgG_1 variants and the His-tagged bovine FcγR proteins were established with the use of Expi293 Expression System (Thermo Fisher Scientific, Waltham, MA, USA). Briefly, Expi293F cells were transfected with pDC6-Boch5D2 IgG_1 WT, pDN112-Boch5D2 IgG_1 ADCC−, pCXN2.1(+)-BoFcγRI-His, pCXN2.1(+)-BoFcγRII-His, pCXN2.1(+)-BoFcγRIII-His, and pCXN2.1(+)-BoFcγ2R-His with the use of Expifectamine (Thermo Fisher Scientific) and cultivated with shaking in Expi293 medium (Thermo Fisher Scientific) at 37°C and 125 rpm with 8% CO_2 for 5–7 days.

Purification of the Boch5D2 IgG_1 variants from the culture supernatant was performed by affinity chromatography and size exclusion chromatography as described in the Materials and Methods. The His-tagged bovine FcγR proteins were purified from the culture supernatants with TALON Metal Affinity Resin (Clontech, Palo Alto, CA, USA), and the buffer was exchanged with PBS by ultrafiltration using Amicon Ultra-15 Centrifugal Filter Unit (Merck Millipore, Billerica, MA, USA). The protein concentrations were measured by ultraviolet absorbance at 280 nm with a NanoDrop 8000 Spectrophotometer (Thermo Fisher Scientific). The protein purities were confirmed by sodium dodecyl sulfate-polyacrylamide gel electrophoresis (SDS-PAGE) as described in the Materials and Methods with a modification to use a SuperSep Ace 5%–20% gradient polyacrylamide gel (Wako Pure Chemical Industries, Osaka, Japan).

Binding assay of Boch5D2 IgG_1 variants to bovine FcγRs

To assess binding of Boch5D2 IgG_1 WT and ADCC− to bovine FcγRs, ELISAs were performed with BoFcγRI-His, BoFcγRII-His, BoFcγRIII-His, and BoFcγ2R-His proteins. Boch5D2 IgG_1 WT or ADCC− was diluted at various concentrations (50, 25, 12.5, 6.25, 3.12, 1.56 nM in 0.05 M carbonate-bicarbonate buffer (Sigma-Aldrich, St. Louis, MO, USA) and coated onto Nunc MaxiSorp ELISA plates (Nunc, Roskilde, Denmark) at 37°C for 2 h. The plates were washed with PBS supplemented with 0.05% Tween20 (PBS-T) and incubated with SuperBlock (PBS) Blocking Buffer (Thermo Fisher Scientific) 37°C for 30 min. After washing with PBS-T, BoFcγRI-His, BoFcγRII-His, BoFcγRIII-His, or BoFcγ2R-His protein diluted to 10 μg/ml was incubated at 37°C for 1 h. The plates were washed again with PBS-T, and FcγR bindings to antibody were detected by anti-polyhistidine tag mouse monoclonal antibody (Abcam, Cambridge, England, UK), HRP-conjugated anti-mouse IgG goat polyclonal antibody (MP Biomedicals), and TMB One Component Substrate (Bethyl Laboratories). The anti-mouse IgG secondary antibody was confirmed no cross-reactivity to bovine IgG including the Boch5D2 IgG_1 variants (data not shown).

Statistical Analysis

Significant differences were identified by repeated one-way analysis of variance (ANOVA) followed by Dunnett's test. All statistical tests were performed with GraphPad Prism 6 (GraphPad Software, San Diego, CA, USA). Differences were considered statistically significant when $P < 0.05$.

References

57. Tamura K, Stecher G, Peterson D, Filipski A, Kumar S. MEGA6: Molecular evolutionary genetics analysis version 6.0. *Mol Biol Evol* (2013) **30**:2725–2729. doi:10.1093/molbev/mst197

58. Larkin MA, Blackshields G, Brown NP, Chenna R, McGettigan PA, McWilliam H, Valentin F, Wallace IM, Wilm A, Lopez R, Thompson JD, Gibson TJ, Higgins DG. Clustal W and Clustal X version 2.0. *Bioinformatics* (2007) **23**:2947–2948. doi: 10.1093/bioinformatics/btm404

59. Biasini M, Bienert S, Waterhouse A, Arnold K, Studer G, Schmidt T, Kiefer F, Cassarino TG, Bertoni M, Bordoli L, et al. SWISS-MODEL: Modelling protein tertiary and quaternary structure using evolutionary information. *Nucleic Acids Res* (2014) **42**:252–258. doi:10.1093/nar/gku340

60. Saphire EO, Parren PW, Pantophlet R, Zwick MB, Morris GM, Rudd PM, Dwek RA, Stanfield RL, Burton DR, Wilson IA. Crystal structure of a neutralizing human IgG against HIV-1: a template for vaccine design. *Science* (2001) **293**:1155–1159. doi:10.1126/science.1061692

61. Guex N, Peitsch MC. SWISS-MODEL and the Swiss-PdbViewer: An environment for comparative protein modeling. *Electrophoresis* (1997) **18**:2714–2723. doi:10.1002/elps.1150181505

62. Tahara H, Suzuki Y, Yamamoto K, Kitahara Y, Suzuki Y, inventor; Hokkaido University, Fuso Pharmaceutical Industries Ltd., assignee. *Expression vector for producing protein derived from foreign gene in large quantity using animal cells, and use thereof.* United States patent US 20120122083 (2012)

Figure legends

Figure S1. Kinetics of anti-PD-1 rat mAb 5D2 in serum of the inoculated cow.

A BLV-infected cow ($n = 1$) was inoculated with anti-bovine PD-1 rat mAb (5D2; 0.05 mg/kg). The serum concentration of 5D2 was determined by ELISA precoated with BoPD-1-His protein. Each dot represents the mean of three independent experiments.

Figure S2. Effect of *in vivo* treatment of anti-PD-1 rat mAb 5D2.

(A) IFN-γ production specific for BLV antigen stimulation. PBMCs were cultured with BLV gp51 antigen peptides or control peptide for 1 day. IFN-γ in supernatant was measured using ELISA. (B) Provirus copy number per 50ng DNA of PBMCs from an inoculated cattle. Proviral loads of BLV were quantified in PBMCs at each time point by real-time genomic PCR targeting the BLV *tax* gene. Each dot represents the mean of three independent experiments. Significant differences were determined using a Dunnett's multiple-comparison test across the time points.

Figure S3. Prediction of FcγR binding sites of bovine IgG_1.

(A) Alignment of the amino acid sequences of constant regions of bovine and human IgG_1 (*IGHG1*; GenBank accession numbers CAA44699 and AAC82527, respectively) and their ADCC-reduced (ADCC−) variants. Dots indicate identity to the sequence from bovine *IGHG1* and dashes indicate gaps in the sequence alignment. Domains of bovine IgG_1 are shown in the figure (CH1, 1–96; Hinge, 97–109; CH2, 110–219; CH3, 220–329). FcγRI binding sites of human IgG_1 are indicated in the box. Diamonds indicate mutated or deleted residues in bovine IgG_1 ADCC−. (B) Predicted structure of the constant region

of bovine IgG_1 (GenBank accession number CAA44699) by automated homology modeling using SWISS-MODEL. CH1, hinge, CH2, and CH3 regions are presented in blue, yellow, green, and orange ribbons, respectively. The estimated FcγR binding residues (113E, 114L, 115P, and 116G in light to dark blue; 207G, 210A, and 211P in light to dark red) are presented in sphere model.

Figure S4. Establishment of Boch5D2 IgG_1 variants and soluble bovine FcγRs.

Production and purification of Boch5D2 IgG_1 WT and ADCC− (A) and BoFcγRI-His, BoFcγRII-His, BoFcγRIII-His, and BoFcγ2R-His (B). These proteins were purified from supernatants of shaking cultures. Purified proteins were confirmed by reducing or nonreducing SDS-PAGE.

Figure S5. Binding of Boch5D2 IgG_1 variants to soluble bovine FcγRs.

The bindings of BoFcγRI-His (A), BoFcγRII-His (B), BoFcγRIII-His (C), and BoFcγ2R-His (D) to Boch5D2 IgG_1 variants were determined by ELISA precoated with Boch5D2 WT or ADCC− in serial dilutions (50 nM to 1.56 nM).

Figure S6. Establishment of soluble bovine PD-1 protein.

Production and purification of BoPD-1-His. BoPD-1-His was purified from supernatants of shaking cultures. Purified protein was confirmed by reducing SDS-PAGE.

Table S1. Primers used in this study.

Target gene	Primer name	Primer sequence (5'–3')[a]	Amplicon size (bp)	Restriction enzyme	GenBank accession number	Reference
Rat *IGHG2a*	RACE RAG2a-1	ACAAGGATTGCATTCCCTTGG	>800	-	L22652	35
	RACE RAG2a-2	CTCAATTTTCTTGTCCACCTTGGTGC	>800	-	L22652	35
Rat *IGKC*	RACE RACK-1	CTCATTCCTGTTGAAGCTCTTGACGAC	>720	-	L22653	35
	RACE RACK-2	CTCATTCCTGTTGAAGCTCTTGACGACGGG	>720	-	L22653	35
Bovine *PDCD1*	BoPD1 F	ATAAGAATGCG**GCCGCCACCATG**GGGACCCCGCGGGCGC	563	*Not*I	AB510901	This study
	BoPD1 R	GCCCTCGAGTTA*ATGGTGATGGTGATGGTG*GATGACCAGGCTCTGCATCT		*Xho*I		
Bovine *IGHG1*	BoIgG1 CH1 F	CTAGCTAGCACCACAGCCCCGAAAGTCT	990	*Nhe*I	X62916	This study
	BoIgG1 CH3 R	TGCTCTAGATTATTTACCCGCAGACTTAGA		*Xba*I		
Bovine *FcγRI*	BoFcγRI F	ATAAGAATGCG**GCCGCCACCATG**TGGCTCATAATAGCTCT	914	*Not*I	NM_174538	This study
	BoFcγRI R	GCCCTCGAGTTA*ATGGTGATGGTGATGGTG*AGGAGTTGTTGACTGGAGGC		*Xho*I		
Bovine *FcγRII*	BoFcγRII F	ATAAGAATGCTA**GCCACCATG**GGGATCCCCTCATTCCT	719	*Nhe*I	NM_174539	This study
	BoFcγRII R	GCCGATATCTTA*ATGGTGATGGTGATGGTG*CGATGAGGGGCCGCTCGAGC		*Eco*RV		
Bovine *FcγRIII*	BoFcγRIII F	ATAAGAATGCG**GCCGCCACCATG**TGGCAACTGCTACCACC	671	*Not*I	NM_001077402	This study
	BoFcγRIII R	GCCCTCGAGTTA*ATGGTGATGGTGATGGTG*GTGCCAAGGTAGAAAGAATG		*Xho*I		
Bovine *Fcγ2R*	BoFcγ2R F	ATAAGAATGCG**GCCGCCACCATG**GCCCCCACCCTCCCTGCCTTGCTCT	740	*Not*I	NM_001001138	This study
	BoFcγ2R R	GCCCTCGAGTTA*ATGGTGATGGTGATGGTG*ATTCTGCATCGTGTAGTCTG		*Xho*I		

[a] Underlined, restriction enzyme cleavage site; **Bold,** KOZAK sequence; *Italic*, 6 × histidine (His) tag.

Figure S1

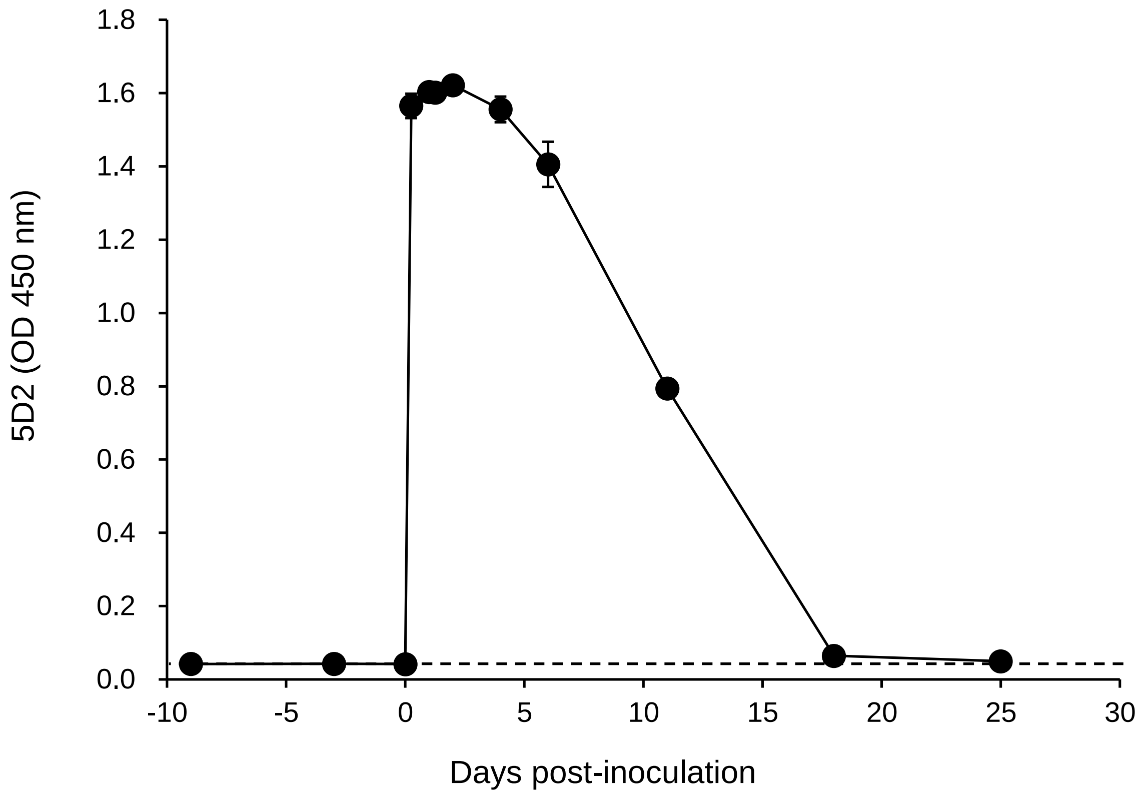
1.8
1.6
1.4
1.2
1.0
0.8
0.6
0.4
0.2
0.0
5D2 (OD 450 nm)
-10
-5
0
5
10
15
20
25
30
Days post-inoculation

Figure S2

A

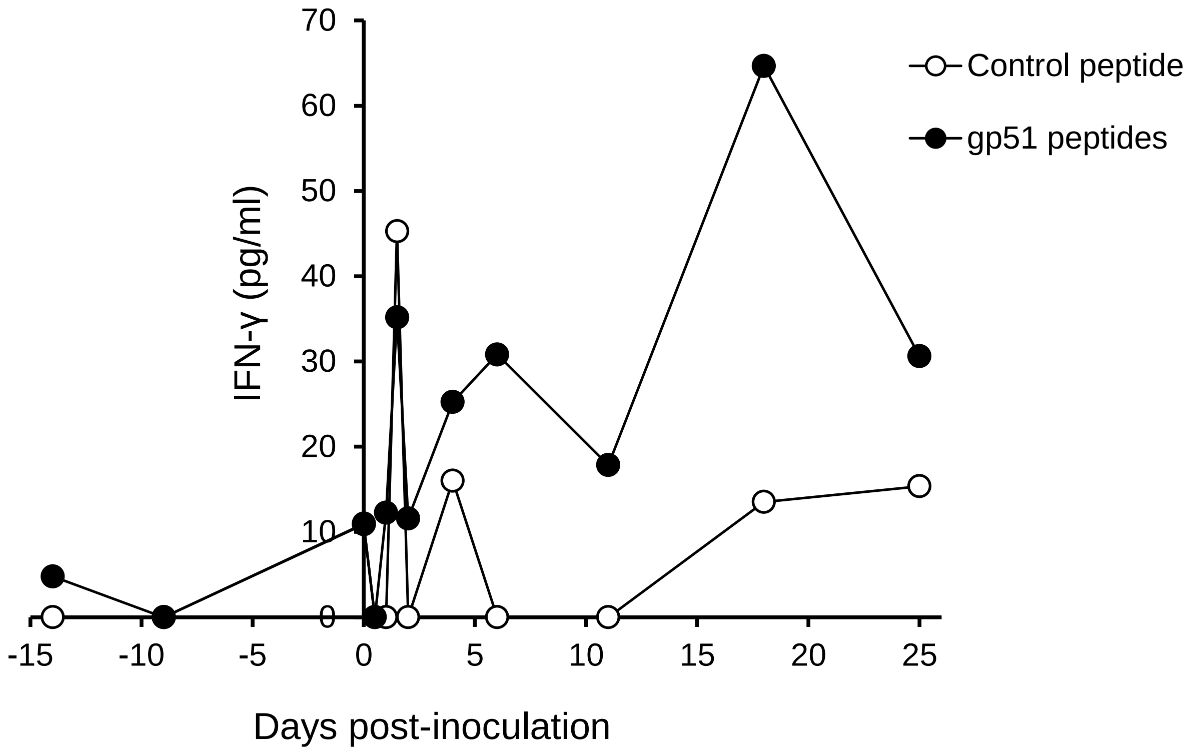

Control peptide
gp51 peptides
IFN-γ (pg/ml)
70
60
50
40
30
20
10
0
-15
-10
-5
0
5
10
15
20
25
Days post-inoculation

B

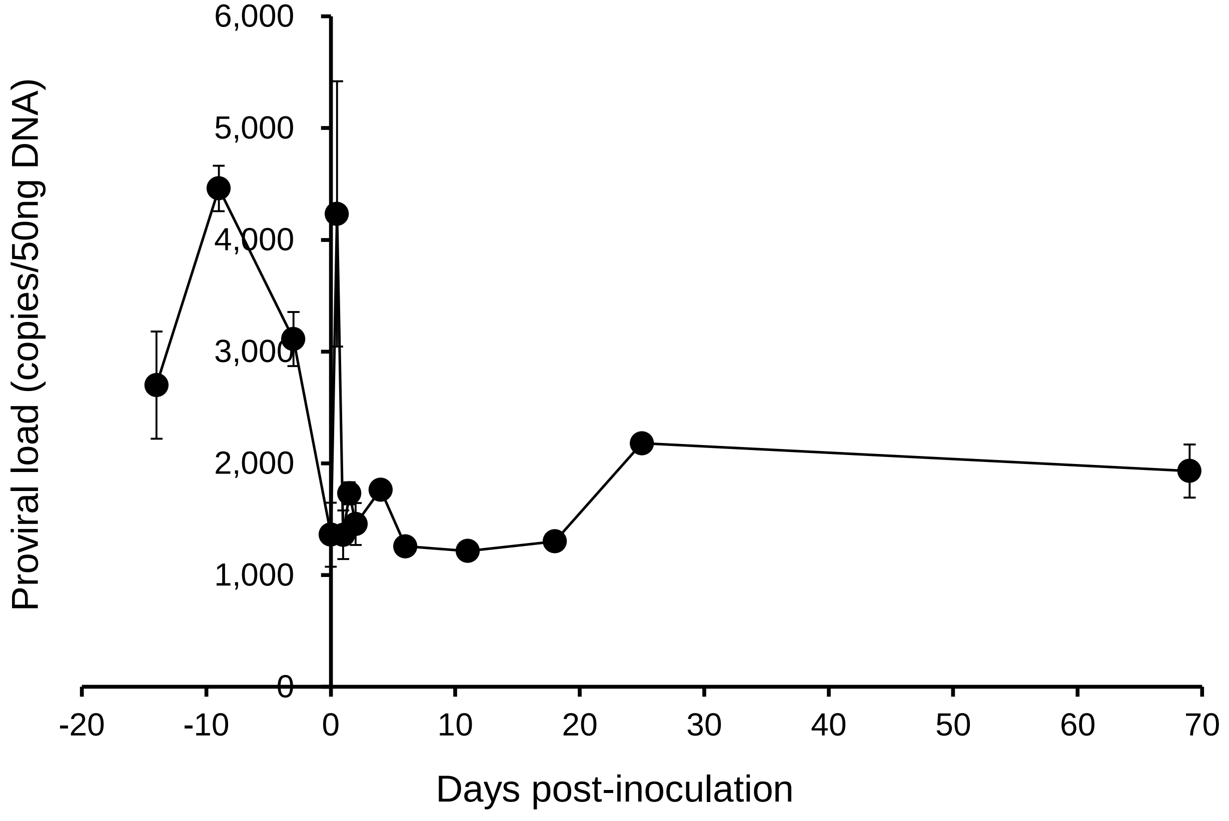

Proviral load (copies/50ng DNA)
6,000
5,000
4,000
3,000
2,000
1,000
0
-20
-10
0
10
20
30
40
50
60
70
Days post-inoculation

Figure S3

A

```
                       |<-------------------------------- CH1 -------------------------------->|<--
Bovine IGHG1         ASTTAPKVYPLSSCCGDKSSSTVTLGCLVSSYMPEPVTVTWNSGALKSGVHTFPAVLQSSGLYSLSSMVTVPGSTSG–QTFTCNVAHPASSTKVDKAVDP   99
Bovine IGHG1 ADCC–   .............................................................................–......................   99
Human IGHG1          ...KG.S.F..APSSKST.GG.AA.....KD.F......S......T....................V....S.SL.T..YI...N.KP.N.....K.E.  100
Human IGHG1 ADCC–    ...KG.S.F..APSSKST.GG.AA.....KD.F......S......T....................V....S.SL.T..YI...N.KP.N.....K.E.  100

                     --- Hinge -->|<------------------------------- CH2 ---------------------------------------
                                    ◆◆◆◆
Bovine IGHG1         –TC–KPSPCDCCPPPELPGGPSVFIFPPKPKDTLTISGTPEVTCVVVDVGHDDPEVKFSWFVDDVEVNTATTKPREEQFNSTYRVVSALRIQHQDWTGGK  197
Bovine IGHG1 ADCC–   –..–...........PVA–.................................................................................  196
Human IGHG1          KS.D.THT.PP..A....L.....L.........M..R...........S.E......N.Y..G...HN.K.......Y........V.TVL....LN..  200
Human IGHG1 ADCC–    KS.D.THT.PP..A.PVA–.....L.........M..R...........S.E......N.Y..G...HN.K.......Y........V.TVL....LN..  199

                     ----------------------->|<-------------------------------- CH3 ------------------------------------
                                 ◆◆
Bovine IGHG1         EFKCKVHNEGLPAPIVRTISRTKGPAREPQVYVLAPPQEELSKSTVSLTCMVTSFYPDYIAVEWQRNGQPESEDKYGTTPPQLDADSSYFLYSKLRVDRN  297
Bovine IGHG1 ADCC–   ............SS......................................................................................  296
Human IGHG1          .Y....S.KA.....EK...KA..QP......T.P.SRD..T.NQ.....L.KG...SD.....ES.....--NN.K....V..S.G.F......T..KS  298
Human IGHG1 ADCC–    .Y....S.K...SS.EK...KA..QP......T.P.SRD..T.NQ.....L.KG...SD.....ES.....--NN.K....V..S.G.F......T..KS  297

                     ------------------------------>|
Bovine IGHG1         SWQEGDTYTCVVMHEALHNHYTQKSTSKSAGK  329
Bovine IGHG1 ADCC–   ................................  328
Human IGHG1          R..Q.NVFS.S..............L.L.P..  330
Human IGHG1 ADCC–    R..Q.NVFS.S..............L.L.P..  329
```

B

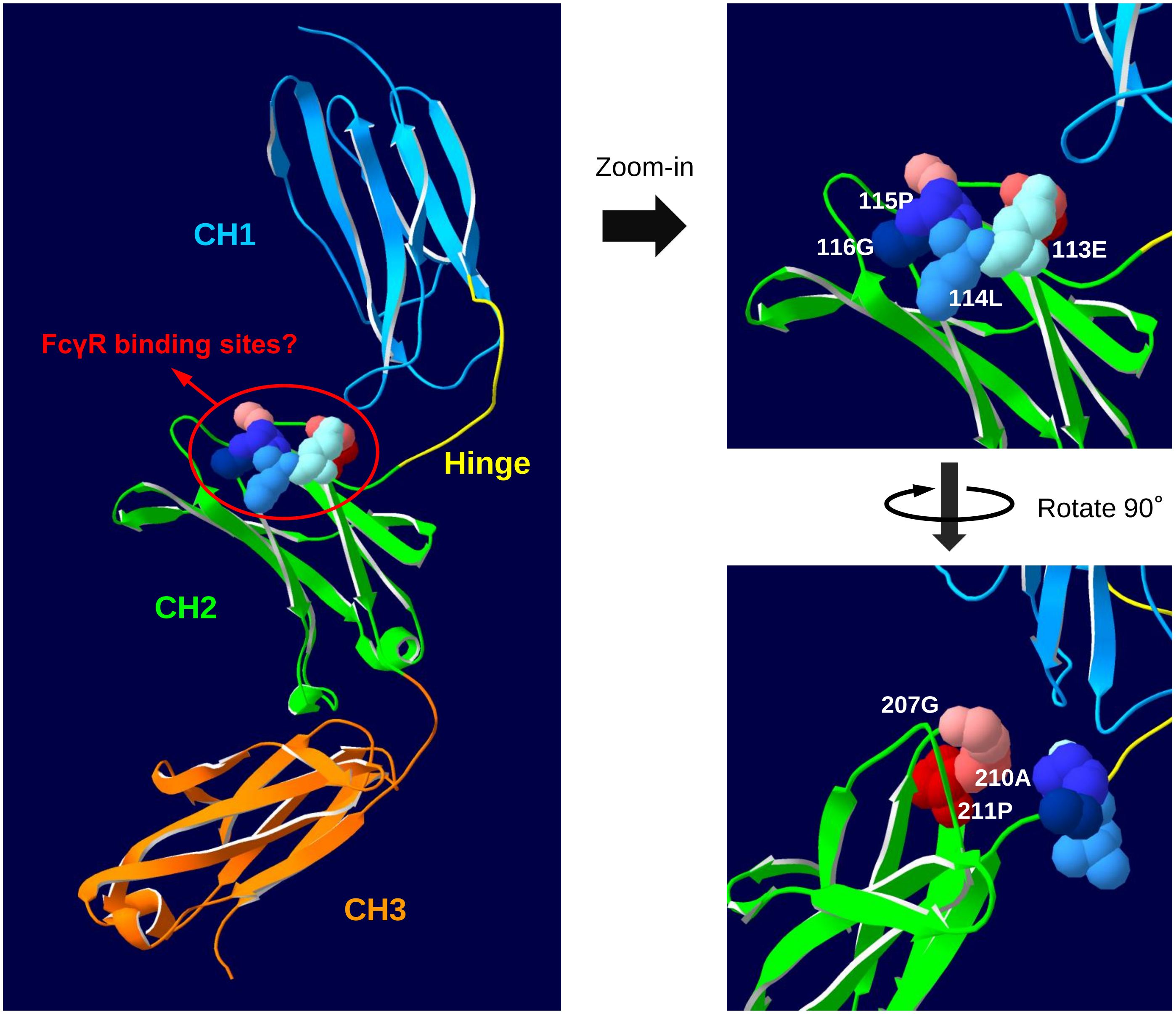

Figure S4

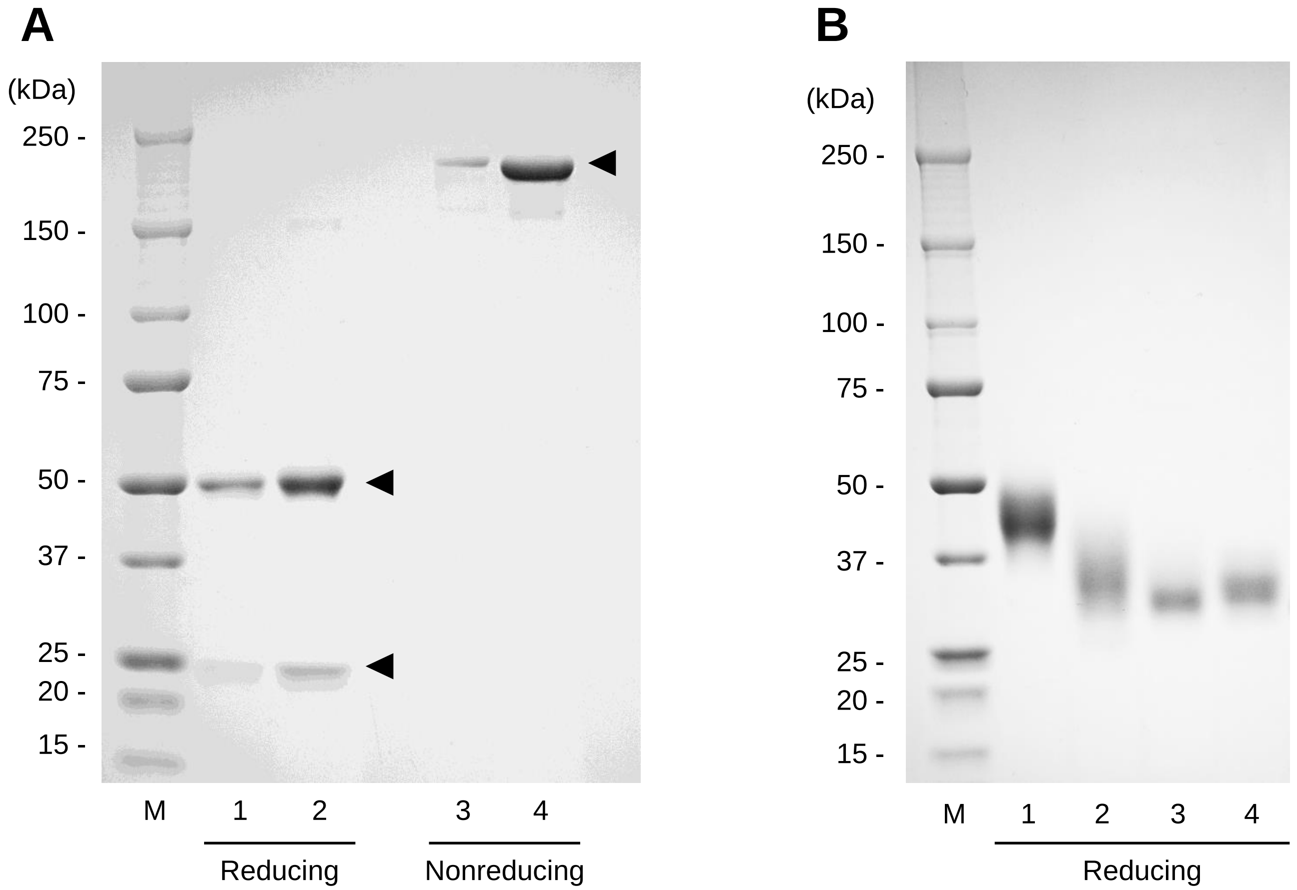

M: Marker
1, 3: Boch5D2 IgG1 WT
2, 4: Boch5D2 IgG1 ADCC−

M: Marker
1: BoFcγRI-His
2: BoFcγRII-His
3: BoFcγRIII-His
4: BoFcγ2R-His

Figure S5

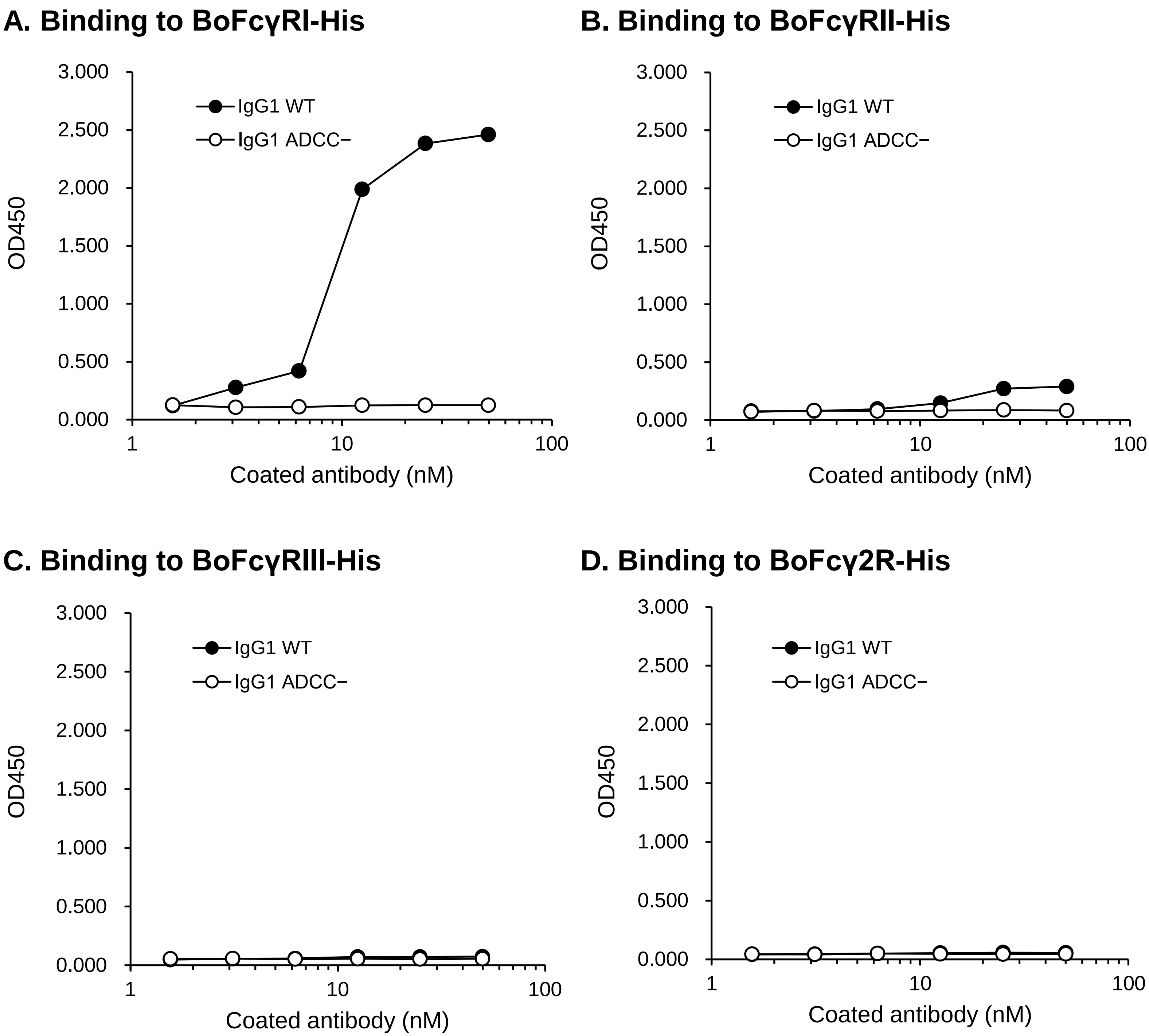
A. Binding to BoFcγRI-His
B. Binding to BoFcγRII-His
C. Binding to BoFcγRIII-His
D. Binding to BoFcγ2R-His
IgG1 WT
IgG1 ADCC−
OD450
Coated antibody (nM)
0.000
0.500
1.000
1.500
2.000
2.500
3.000
1
10
100

Figure S6

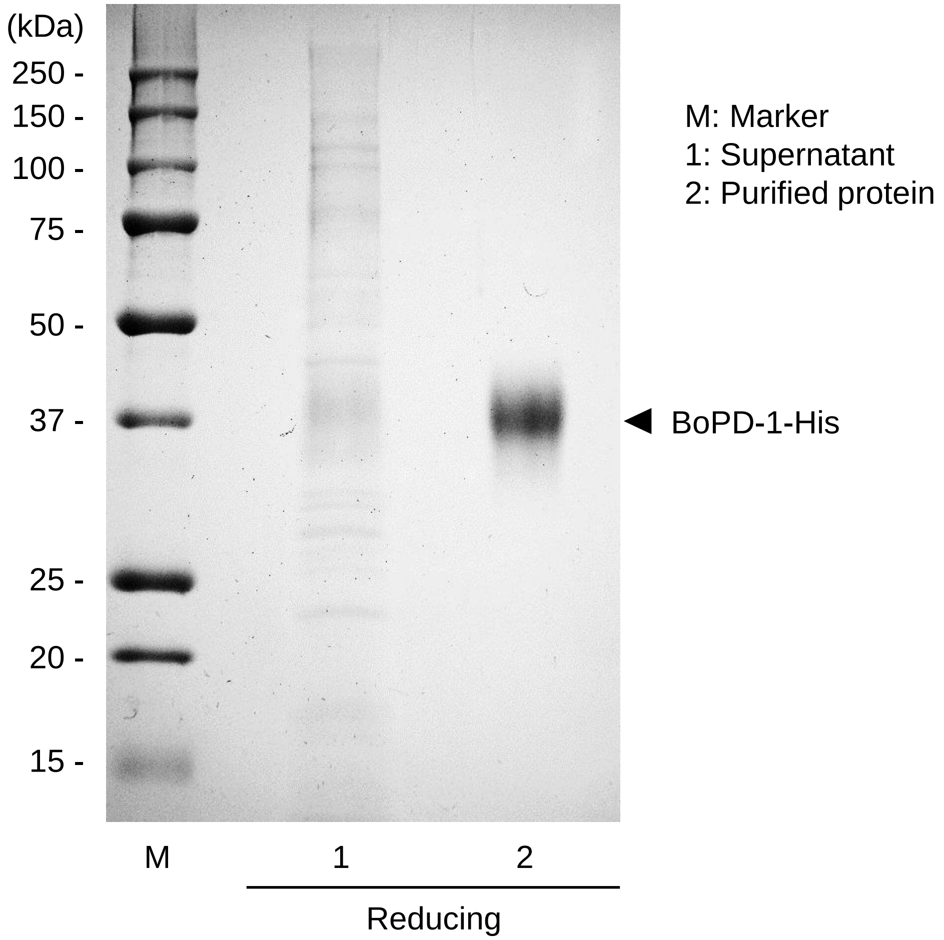
(kDa)
250 -
150 -
100 -
75 -
50 -
37 -
25 -
20 -
15 -
M: Marker
1: Supernatant
2: Purified protein
BoPD-1-His
M
1
2
Reducing
